# Apolipoprotein E Isoform-specific changes related to stress and trauma exposure

**DOI:** 10.1038/s41398-022-01848-7

**Published:** 2022-03-28

**Authors:** Eileen Ruth S. Torres, Jenny Luo, James K. Boehnlein, Daniel Towns, J. David Kinzie, Andrea E. DeBarber, Jacob Raber

**Affiliations:** 1 Department of Behavioral Neuroscience, Oregon Health & Science University, 3181SW Sam Jackson Park Road, L470, Portland, OR 97239 USA; 2 Department of Chemical Physiology & Biochemistry, Oregon Health & Science University, Portland, OR 97239 USA; 3 Department of Psychiatry, Oregon Health & Science University, 3181 S.W. Sam Jackson Park Road, UHN-80, Portland, OR 97201-3098 USA; 4 VA Northwest Mental Illness Research, Education and Clinical Center (MIRECC), Washington DC, USA; 5 Departments of Neurology, Psychiatry, and Radiation Medicine and Division of Neuroscience, ONPRC, Oregon Health & Science University, Portland, OR 97239 USA

**Keywords:** Hippocampus, Depression

## Abstract

Post-Traumatic Stress Disorder (PTSD) is a highly prevalent mental health disorder. Due to the high level of variability in susceptibility and severity, PTSD therapies are still insufficient. In addition to environmental exposures, genetic risks play a prominent role and one such factor is *apolipoprotein E*. The protein (apoE) is functionally involved in cholesterol transport and metabolism and exists as 3 major isoforms in humans: E2, E3, and E4. To model the role of apolipoprotein E isoform in stress-related changes in behavior and cognition, female and male mice (3–5 months of age) expressing E2, E3, or E4 were used. Mice were either placed into control groups or exposed to chronic variable stress (CVS), which has been shown to induce PTSD-like behavioral and neuroendocrine changes. E2 mice showed a unique response to CVS compared to E3 and E4 mice that included impaired spatial learning and memory, increased adrenal gland weight, and no increase in glucocorticoid receptor protein levels (normalized to apoE levels). In addition, the cholesterol metabolite 7-ketocholesterol was elevated in the cortex after CVS in E3 and E4, but not E2 female mice. E2 confers unique changes in behavioral, cognitive, and biomarker profiles after stress exposure and identify 7-ketocholesterol as a possible novel biomarker of the traumatic stress response. We further explored the relationship between E2 and PTSD in an understudied population by genotyping 102 patients of Cambodian and Vietnamese ethnicity. E2 carriers demonstrated a higher odds ratio of having a PTSD diagnosis compared to E3/E3 carriers, supporting that the E2 genotype is associated with PTSD diagnosis after trauma exposure in this population.

## Introduction

Post-Traumatic Stress Disorder (PTSD) is diagnosed in 7.8% of the population although most individuals will experience trauma at some point in their lifetime [1]. Symptoms are categorized into re-experiencing trauma, negative alterations in cognitions and mood, altered arousal and reactivity, and avoidance (DSM V). Many patients suffer comorbid conditions, including depression and anxiety, and cardiovascular disease [2, 3] that range widely in severity and endure for decades [4], presenting heterogeneity that complicates therapies.

Environmental [5] and genetic risk factors [6–8] modulate risk and severity of PTSD. Apolipoprotein E (apoE) exists in 3 major isoforms in humans—E2, E3, and E4—and is a major player in lipid transport and metabolism. E4 is the strongest genetic risk factor of Alzheimer’s disease (AD), especially in women [9], and has been associated with cardiovascular disease [10, 11]. In contrast, E2 is considered protective for AD compared to E3 [9]. Both E4 [12–14] and E2 [15–17] have been suggested to be associated with PTSD symptom severity and susceptibility. Due to the relative lower ε2 allelic frequency [18], E2 is often precluded from analyses. Moreover, these studies often have focused on men, yet women are more likely to develop PTSD [19]. Thus, the relationship between *APOE* genotype and PTSD is still not yet fully understood.

Chronic variable stress (CVS) exposure is used to model PTSD-related symptoms and underlying mechanisms in rodents [20, 21]. Previously, the association between PTSD-related symptoms and different human apoE isoforms was assessed by comparing performance of male human apoE targeted replacement (TR) mice in fear conditioning and CVS paradigms. E2, but not E3 or E4, mice demonstrated impaired fear extinction learning [16, 22] and CVS exposure led to unique behavioral and neuroendocrine changes associated with PTSD in E2 mice [16].

These E2 effects might involve the low-density lipoprotein receptor (LDLR), the major apoE receptor in the CNS [23]. E2 has a lower binding affinity (~1%) to LDLR than E3 and E4, which is normally compensated for by other apoE-binding receptors in the LDLR family [24]. Subsequent cholesterol transport and metabolism involving apoE and LDLR may result in changes in the utilization and metabolism of cholesterol. Unlike cholesterol, oxysterols, oxidized metabolites of cholesterol, can penetrate the blood-brain barrier and act as signaling molecules for cholesterol metabolism [25]. One such oxysterol, 7-ketocholesterol, inhibits glucocorticoid action in adipocytes [26] and serves as a ligand for oxysterol binding protein receptors, which attenuate glucocorticoid synthesis, in the adrenal gland [27]. In brain, 7-ketocholesterol levels increase with disease progression in the frontal cortex of AD patients [28].

To increase current understanding of apoE genotype after stress exposure, we examined sex differences interacting with apoE genotype in behavioral and cognitive performance differences associated with CVS, LDLR levels, components of the HPA axis (GR and corticosterone), and developed an assay to measure sterols and oxysterols in low volumes of murine tissue to analyze cholesterol precursors and metabolites. Finally, we assessed if APOE genotype was also relevant in a clinical non-Caucasian population.

Specifically, we genotyped male and female Cambodian and Vietnamese refugees in the Portland area receiving care through the Oregon Health & Science University (OHSU) Intercultural Psychiatry Program (IPP), a long-standing, cross-cultural clinic in the community [29].

## Methods

### Animals and experimental design

**Fig. 1:**
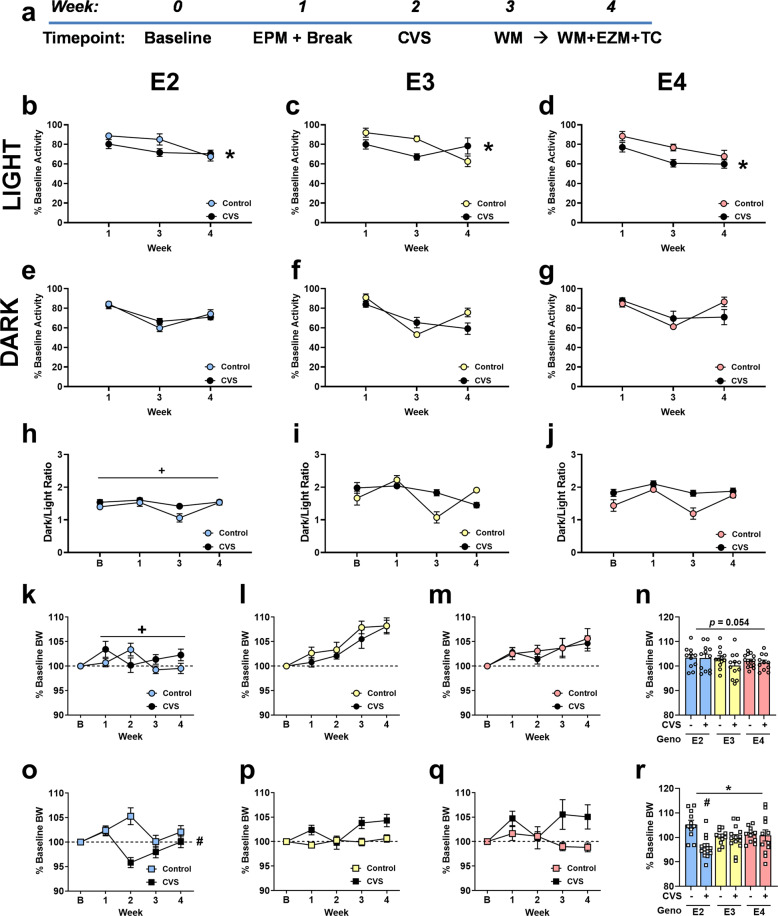
Experimental design and home cage activity. **a** Experimental design, body weights, and home cage activity. EPM = Elevated Plus Maze. CVS = Chronic Variable Stress. WM = Water Maze. EZM = Elevated Zero Maze. TC = Tissue Collection. **b**–**d** Percent baseline home cage activity during the light cycle throughout the experiment shows that CVS exposure resulted in overall less activity compared to controls (*p < 0.05). **e**–**g** Percent baseline home cage activity during the dark cycle was not altered by genotype or CVS exposure. **h**–**j** Activity was also assessed a ratio of activity during the light and dark cycles, which showed E2 mice overall had lower dark/light ratios (+*p* < 0.05). B = Baseline. Sexes were collapsed for panels **b**–**j**. **k**–**m** Female mice overall gained body weight throughout the experiment; however, this growth was not seen in either female E2 group (+*p* < 0.05). **o**–**q** In males, percent baseline bodyweights changed throughout the weeks of testing. These within-subject changes over the weeks were influenced by genotype and CVS exposure, as well as by an interaction between genotype and CVS exposure (# *p* < 0.05). Percent baseline bodyweight during week 2, the week of CVS exposure, was assessed separately to better understand these interactions. While females exposed to CVS showed a trend to lower percent baseline bodyweights (*p* = 0.054) (**n**), CVS exposed males showed lower percent baseline bodyweights compared to controls (*p < 0.05) which was due to the lower percent baseline bodyweight in E2 CVS males compared to E2 control males (**r**) genotype x group interaction: #*p* < 0.05. Symbols: + refers to pairwise comparison of genotype effect, * refers to CVS effect, # refers to genotype x CVS interaction.

All housing and experimental procedures were approved by the OHSU Institutional Animal Care and Use Committee (IACUC). Male and female human apoE TR mice, models originally generated by Dr. Patrick Sullivan [30–32] and 3–5 months of age at the start of the experiments, were included. Food and water were available *ad libitum* except as noted. Lights in the housing room were set to 12 hr light: 12 hr dark cycle. All behavioral tests and procedures took place during the light phase, except for home cage activity which occurred continuously. Figure 1a summarizes the experimental design. For group sizes, behavioral testing, plasma and tissue collection and preparation, plasma corticosterone analysis, protein and lipid analyses, subject enrollment, saliva sample collection and genotyping, and statistical analyses, see Supplementary Table 1, Supplementary Figs. 1, 2, and the Supplemental Information.

## Results

### Home cage activity and body weights

Due to the impact of PTSD symptoms such as nightmares and altered arousal on patient quality of life, we assessed home cage activity as a measure of circadian activity throughout the experiment (Fig. 1a). Light and dark phases were analyzed as separate outcomes. Due to sex, genotype, and CVS-exposure group differences during the baseline week (Supplementary Fig. 3), activity was further assessed by normalizing to baseline activity (Fig. 1b–g). Since CVS exposure disrupted only the CVS groups, Week 2 was excluded from analysis. During the light phase, mice showed decreased activity (*F*_1.74, 78.13_ = 10.54, *p* < 0.001), suggesting habituation to the change in housing conditions, and a sex x genotype interaction (*F*_3.48, 69.60_ = 2.88, *p* = 0.035); E2 females showed less habituation than E2 males. CVS-exposed mice were less active than controls (*F*_1, 40_ = 7.68, *p* = 0.008). During the dark phase, there was a decrease in activity during Week 3, but an increase in activity at the next time point (*F*_1.50, 59.87_ = 89.24, *p* < 0.001). This was influenced by genotype; activity in E3 mice remained lower during Week 4 (Week x Genotype interaction: *F*_2.99, 59.89_ = 3.98, *p* = 0.012).

The ratio of dark to light activity (Fig. 1hi, j) was analyzed as well. There was an effect of week (*F*_2.28, 104.90_ = 9.81, *p* = 0.001), a week x genotype interaction (*F*_4.56, 104.90_ = 3.37, *p* = 0.009), and a week x CVS condition interaction (*F*_2.28, 104.90_ = 3.447, *p* = 0.030). Activity level in CVS groups did not vary across weeks, whereas activity levels in the controls decreased during Week 3 and recovered by Week 4. There was a trend towards E2 mice exposed to CVS showing less change in dark phase activity than E2 controls (trend towards a week × genotype × group interaction: *F*_4.56, 104.90_ = 2.092, *p* = 0.078). E2 mice also showed lower dark/light ratios overall (*F*_2, 46_ = 22.942, *p* < 0.001: pairwise comparisons E2 vs E3: *p* < 0.001, E2 vs E4: *p* < 0.001). Thus, CVS exposure altered activity during the light phase and E2 mice demonstrated lower overall activity, *i.e*. dark/light ratio, than E3 and E4 mice.

People with PTSD also tend to have problems with weight gain and obesity [33]. Middle-aged male apoE deficient (knockout (KO)) mice show increased food intake and body weights and decreased fat deposits [34], suggesting a potential role for apoE. Males weighed more than females at baseline (Supplementary Fig. 4), which was influenced by genotype and CVS exposure group. Consequently, we analyzed % baseline body weight over the rest of the experiment. Males and females showed different changes in % baseline body weight over the course of the 5 weeks of the experiment (Fig. 1k–r. Week x sex interaction: *F*_2.97, 410.43_ = 7.29, *p* < 0.001) as well as overall different % baseline bodyweights (*F*_1, 138_ = 6.90, *p* = 0.010) and were hence assessed separately.

Females grew in body weight throughout the experiment (Fig. 1k–m. *F*_3.08, 206.37_ = 21.59, *p* < 0.001), which was influenced by genotype (*F*_6.16, 206.37_ = 8.89, *p* < 0.001). E2 mice showed the least growth over time (Fig. 1k. *F*_2, 67_ = 5.85, *p* = 0.005, pairwise comparisons E2 vs E3: *p* = 0.004). Males demonstrated less consistent growth, *i.e*. all groups dramatically shifted during Week 2, (Fig. 1o-q. *F*_2.59, 183.80_ = 7.075, *p* < 0.001) that was affected by genotype (*F*_5.18, 183.80_ = 2.89, *p* = 0.014), CVS condition (*F*_2.59, 183.80_ = 14.15, *p* < 0.001), and a genotype x CVS condition interaction (*F*_5.18, 183.80_ = 3.95, *p* = 0.002). There was also a sex x genotype x CVS condition interaction (*F*_2, 71_ = 5.40, *p* = 0.007). Therefore, we further analyzed the % baseline body weights with the sexes separated during Week 2, which was the week during CVS exposure. Female mice showed no genotype differences, but there was a trend towards decreased weight in CVS groups (Fig. 1n. *F*_1, 67_ = 3.84, *p* = 0.054). Males showed a difference between CVS conditions (Fig. 1r. *F*_1, 71_ = 6.90, *p* = 0.011) and a genotype x group interaction (*F*_2, 71_ = 7.39, *p* = 0.001). These interactions were driven by lower % baseline bodyweight in E2 CVS than E2 control mice.

### Spatial learning and memory

**Fig. 2:**
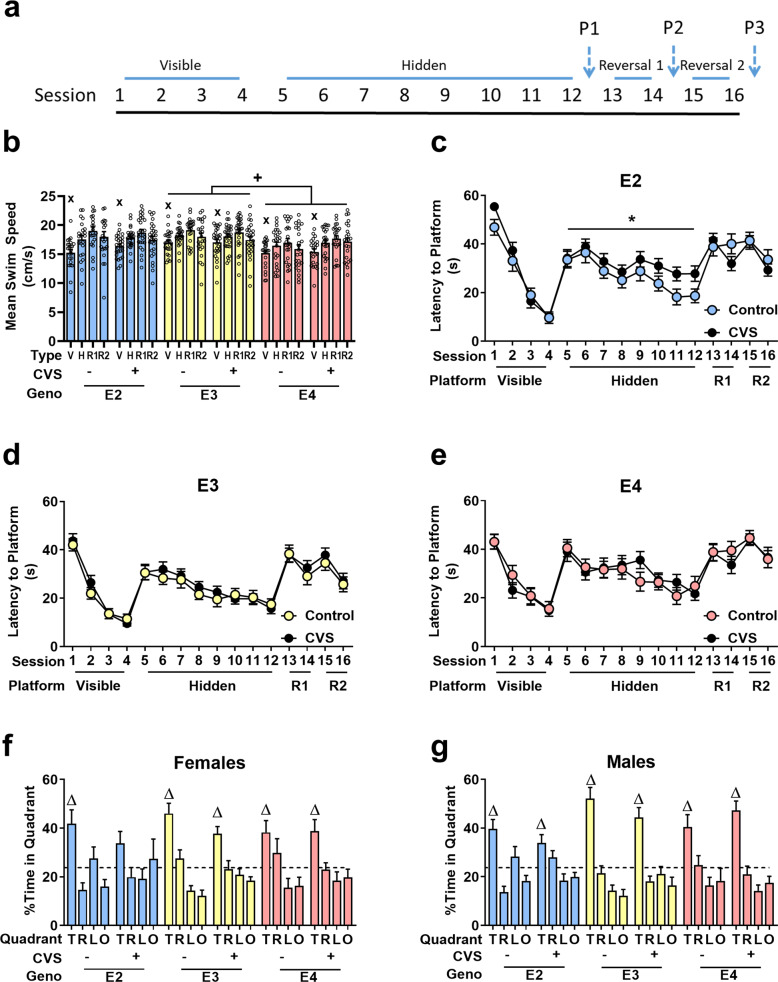
Spatial learning and memory were assessed in the water maze. **a** Timeline of session types over the 9 days of testing. P = Probe. **b** E4 mice swam the slowest throughout testing (+*p* < 0.05). Mice overall swam slower during visible platform trials compared to hidden and reversal trials (^x^*p* < 0.05). V = Visible, H = Hidden, R = Reversal. **c**–**e** Latency to locate the target platform is shown for the different genotypes. Sexes are shown collapsed. E2 mice (**c**) were the only genotype to be significantly affected by CVS throughout any of the sessions. E2 CVS exposed mice improved less over hidden training session compared to controls (*p = 0.05) and E2 control mice did not improve during the first reversal location testing, compared to E2 CVS mice (*p < 0.05). E3 (**d**) and E4 (**e**) mice did not show significant differences due to CVS. **f**, **g** Percent time in each quadrant is shown for probe 1. T = Target, R = Right, L = Left, O = Opposite. Female E2 mice exposed to CVS was the only group that failed to show a preference for the target quadrant (Δ *p* < 0.05). Data for **b**–**e** are shown with sexes collapsed. Symbols: + refers to pairwise comparison of genotype effect, * refers to CVS effect, ^x^ refers to effect of session type, Δ refers to effect of quadrant within subjects.

After all groups were exposed to CVS or the control condition, mice were tested for spatial learning and memory in the water maze. The testing paradigm used is shown in Fig. 2a. Since swim speed can confound cognitive performance measures, average swim speeds were assessed during visible platform, hidden platform, reversal 1 (second hidden platform location) and reversal 2 (third hidden platform location) (Fig. 2b**)** training. Sex was not significant (*F*_1,138_ = 1.744, *p* = 0.189). Therefore, the data in panels **b**–**e** are collapsed across sexes. Session type affected average swim speed (*F*_2.67, 369.029_ = 43.87, *p* < 0.001) with mice faster during hidden and reversal sessions than visible training sessions. E4 mice swam slower than E3 mice (Genotype: *F*_2,138_ = 5.48, *p* = 0.005, pairwise comparisons: E3 vs E4: *p* = 0.004). Swim speed during each session type was used as a covariate when latency to the platform was analyzed. During all types of hidden platform training sessions, there was an effect of genotype (Visible: *F*_2, 137_ = 3.78, *p* = 0.025; Hidden: *F*_2,137_ = 5.590, *p* = 0.005; Reversal 2: *F*_1,137_ = 6.28, *p* = 0.013; Reversal 2: *F*_1,137_ = 5.45, *p* = 0.005).

Further analyses were performed with genotypes separated. During visible platform training, there was a session x sex x CVS condition interaction in E2, but not E3 and E4, mice for the latency to the platform (*F*_2.52,171.118_ = 3.62, *p* = 0.022) (Fig. 2c–e). During hidden platform training sessions of E2 mice, swim speed was a significant covariate (*F*_1,45_ = 4.72, *p* = 0.035). There was also a trending effect of CVS (*F*_1,45_ = 3.96, *p* = 0.053). One E2 female control mouse failed to learn the task and timed out during 29 of the 32 trials, the most in all groups. To see if this individual influenced the data, we removed it and collapsed the sexes. CVS E2 mice showed poorer performance than E2 controls (Fig. 2c. *F*_1,45_ = 4.055, *p* = 0.050). While E3 males did slightly better than E3 females (*F*_1,47_ = 4.064, *p* = 0.050), neither E3 nor E4 mice showed any differences due to CVS exposure. There were no differences during reversal 1 or reversal 2 within each genotype group.

To assess spatial memory retention during the first probe trial, preference to spend time in the target quadrant compared to the three non-target quadrants was analyzed separately for each group (Fig. 2f, g). Except for female E2 CVS mice, all mice showed an effect of quadrant (ANOVA: E2 female controls: *F*_3,30_ = 4.32, *p* = 0.012; E3 female controls *F*_3,33_ = 18.15, *p* < 0.001; E4 female controls *F*_3,33_ = 4.31, *p* = 0.011; E3 female CVS: *F*_3,39_ = 7.78, *p* < 0.001; E4 female CVS: *F*_3,30_ = 4.86, *p* = 0.007; E2 male controls: *F*_3,30_ = 9.41, *p* < 0.001; E3 male controls *F*_3,33_ = 24.36, *p* < 0.001; E4 male controls; *F*_1.71, 18.76_ = 4.60, *p* = 0.028; E2 male CVS: *F*_3,45_ = 5.27, *p* = 0.003; E3 male CVS: *F*_3, 39_ = 12.64, *p* < 0.001; E4 male CVS: *F*_3, 33_ = 17.34, *p* < 0.001). Pairwise comparisons within each group demonstrated that the effect of quadrant was due to more time being spent within the target quadrant except for female E2 CVS mice. Following reversal training, no group showed preference for the new target quadrant (Supplementary Table 2).

### Changes in anxiety-like behavior and related physiological measures

**Fig. 3:**
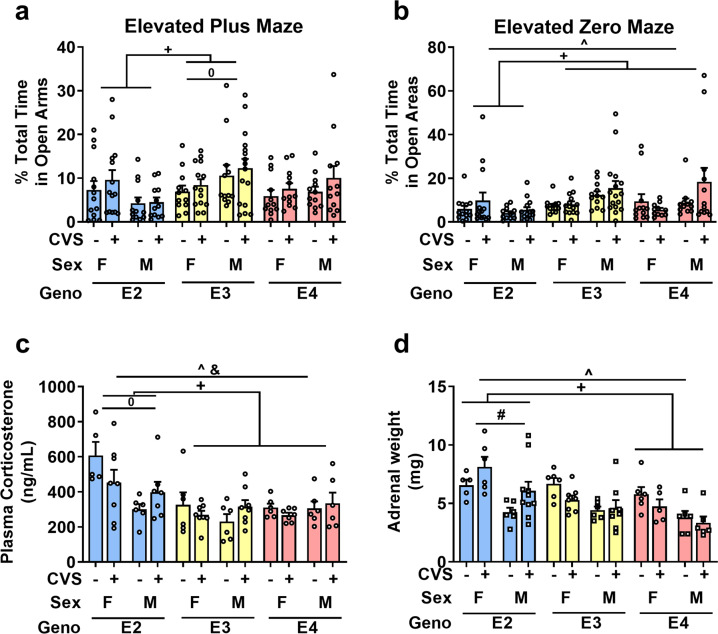
Behavioral and physiological anxiety-related measures. **a** E2 mice spent significantly less time in the open arms of the elevated plus maze compared to E3 mice (+*p* < 0.05). E3 females also spent less time than E3 males (^*p* < 0.05). **b** E2 mice again explored the least time in the open areas of the elevated zero maze (+*p* < 0.05). Males explored the open areas more than females (^*p* < 0.05). **c** Plasma corticosterone levels after a mild stressor (elevated zero maze) showed that females had higher levels compared to males (^*p* < 0.05) which was driven by the difference in E2 mice (^0^*p* < 0.05*)*. E2 mice had the highest corticosterone levels (+*p* < 0.05). There was also an interaction between sex x group driven by the lower corticosterone levels seen in female mice exposed to CVS (^&^*p* < 0.05). **d** Adrenal glands were dissected after all behavioral testing. Females had heavier adrenal glands than males (^*p* < 0.05). E2 mice had the heaviest overall (+*p* < 0.05) and CVS exposed E2 mice had larger glands compared to their genotype-matched controls (*p < 0.05). Symbols: + refers to pairwise comparison of genotype effect, ^ refers to sex effect, * refers to CVS effect, # refers to genotype x CVS interaction, ^0^ refers to sex x genotype interaction, ^&^ refers to sex x CVS interaction.

E2 mice explored the open arms in the elevated plus maze less than E3 mice, suggesting higher anxiety levels (Fig. 3a, *F*_2, 137_ = 6.73, *p* = 0.002; E2 vs E3: *p* = 0.001). Within genotype, sex differences were only seen in E3 mice, with females spending less time in the open arms than males (Sex x genotype interaction: *F*_2, 137_ = 4.011, *p* = 0.020).

After CVS exposure, there was again a difference due to genotype with E2 mice spending less time in the open areas of the zero maze (Fig. 3b, *F*_2, 137_ = 5.75, *p* = 0.004; E2 vs E3: *p* = 0.007, E2 vs E4: *p* = 0.023). Female mice explored the open areas of the zero maze less than males (*F*_1, 137_ = 6.23, *p* = 0.014). CVS exposure did not result in group differences in the time spent in the open areas of the elevated zero maze.

Plasma corticosterone, the major corticosteroid in rodents, was measured immediately after mice completed the elevated zero maze test (Fig. 3c) to assess physiological changes. There was an interaction between sex and CVS condition; female CVS mice had lower plasma corticosterone levels compared to controls whereas male CVS mice had higher plasma corticosterone levels compared to controls (*F*_1, 68_ = 4.82, *p* = 0.032). E2 mice had higher plasma corticosterone levels compared to E3 and E4 (*F*_2, 68_ = 8.70, *p* < 0.001; pairwise comparisons: E2 vs E3 *p* = 0.001, E2 vs E4 *p* = 0.005), and E2 females had significantly higher plasma corticosterone levels than other groups (sex x genotype interaction: *F*_2, 68_ = 5.55, *p* = 0.006). There was also a sex x CVS condition interaction (*F*_1, 68_ = 5.25, *p* = 0.025) driven by decreased levels of corticosterone in female CVS-exposed mice. This suggests that E2 mice respond to an anxiety-provoking maze in a sex-dependent manner.

Following behavioral and cognitive testing, adrenal glands were removed and weighed as an indirect measure of glucocorticoid secretion [35, 36] (Fig. 3d). Females had heavier adrenal glands compared to males (*F*_1, 66_ = 23.67, *p* < 0.001) and E2 mice had larger adrenal glands compared to E4 mice (*F*_2, 66_ = 8.08, *p* = 0.001; pairwise comparison E2 vs E4: *p* < 0.001). Furthermore, E2 mice exposed to CVS showed larger adrenal glands compared to their genotype-matched controls (ANOVA: Genotype x group interaction: *F*_2, 66_ = 4.67, *p* = 0.013).

### Level of target proteins

In the frontal cortex, apoE, LDLR, and GR levels were not significantly different due to sex, genotype, or CVS exposure as analyzed by Western blot **(**Supplementary Table 3**)**.

**Fig. 4:**
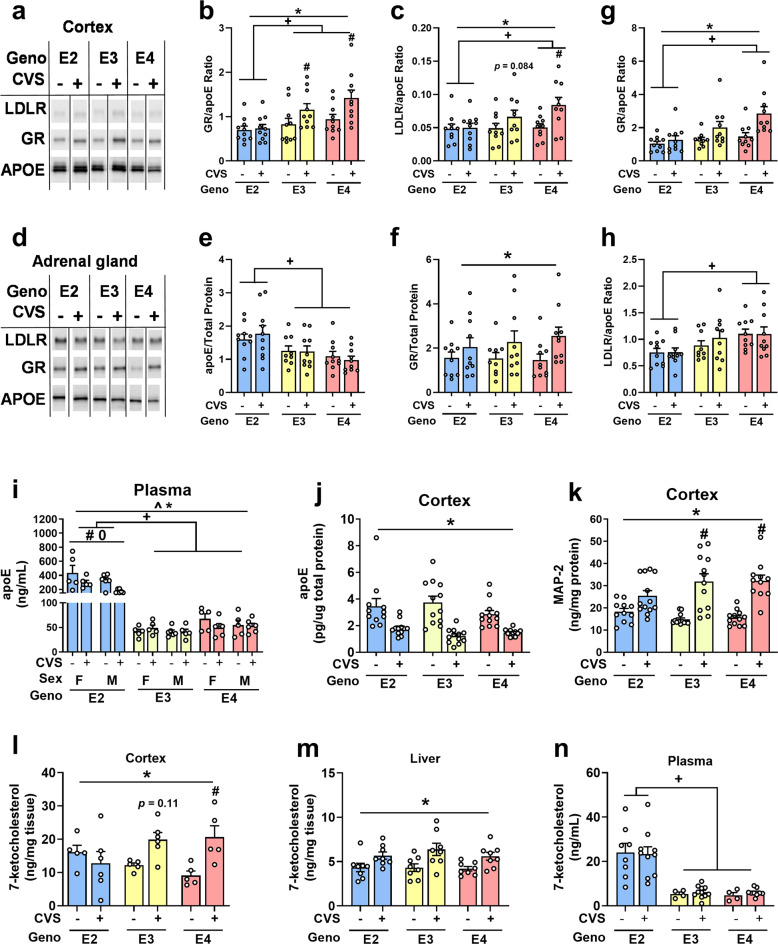
Protein and lipid measurements in tissue and plasma. **a** Example cortex Western blot. **b** E2 mice had the lowest GR/APOE ratio (+*p* < 0.05) and E3 and E4 CVS-exposed mice showed larger ratios compared to controls (#*p* < 0.05). **c** LDLR/apoE ratios had similar differences to the GR/apoE ratios (+*p* < 0.05, #*p* < 0.05). **d** Example adrenal gland Western blot. **e** E2 mice had the highest apoE levels in adr**e**nal glands (+*p* < 0.05). **f** GR in the adrenal gland was higher in CVS-exposed mice (*p < 0.05). **g** GR/apoE ratios were lowest in E2 mice (+*p* < 0.05). CVS-exposure resulted in higher GR/apoE ratio compared to controls (*p < 0.05). **h** E2 mice had the lowest LDLR/apoE ratios (+*p* < 0.05). **i** Plasma levels of apoE were hig**h**est in E2 mice (+*p* < 0.05). Furthermore, E2 mice exposed to CVS had lower apoE levels compared to controls (*p < 0.05). Female E2 mice had more plasma apoE than male E2 mice (^0^*p* < 0.05). **j** In cortical tissue, CVS exposure led to lower apoE levels (*p < 0.05). **k** Meanwhile, MAP-2 levels in the cortex were higher in mice exposed to CVS (*p < 0.05). **l** Female cortical tissue showed a genotype x group interaction in which only E4 mice exposed to CVS showed higher levels of 7-ketocholesterol compared to controls (#*p* < 0.05). **m** CVS exposure was associated with higher 7-ketocholesterol levels (*p < 0.05) regardless of genotype or sex (shown collapsed). **n** Plasma levels were highest in E2 mice (+*p* < 0.05, shown with sexes collapsed). Symbols: + refers to pairwise comparison of genotype effect, * refers to CVS effect, ^ refers to sex effect, # refers to genotype x CVS interaction, 0 refers to sex x genotype interaction.

Since LDLR levels influence apoE and apoE appears to modulate the glucocorticoid system, we normalized both LDLR and GR to apoE protein levels to assess if there was a relationship between these markers and CVS exposure that was dependent on the amount of apoE. There was an apoE isoform-dependent effect on GR/apoE in the cortex (Fig. 4a, b. *F*_2, 47_ = 18.26, *p* < 0.001; pairwise comparisons E2 vs E3: *p* = 0.003; E2 vs E4: *p* < 0.001; E3 vs E4: *p* = 0.052). Furthermore, CVS mice had higher GR/apoE ratios versus controls (*F*_1, 47_ = 19.70, *p* < 0.001) as observed in E3 and E4 mice (genotype x group: *F*_2, 47_ = 4.33, *p* = 0.019). Analyses of LDLR/apoE ratios revealed similar differences in the cortex, specifically that E2 showed the lowest ratios (Fig. 4c. *F*_2,47_ = 4.85, *p* = 0.012: E2 vs E4 *p* = 0.009). In addition, CVS mice had larger ratios compared to control counterparts (*F*_1,47_ = 13.39, *p* = 0.001) which was again absent in E2 mice (*F*_2, 47_ = 3.85, *p* = 0.028).

Western blot analysis of adrenal glands (Fig. 4d) showed apoE levels were highest in E2 mice (Fig. 4e. *F*_2, 46_ = 9.568 *p* < 0.001: pairwise comparisons: E2 vs E3: *p* = 0.018, E2 vs E4: *p* < 0.001). CVS conditions did not show differences in adrenal LDLR levels (Supplementary Table 3). GR levels were higher in CVS exposed mice compared to control mice (Fig. 4f. *F*_1, 46_ = 6.37, *p* = 0.015). GR/apoE ratios were also dependent on apoE isoform (Fig. 4g. *F*_2, 46_ = 8.17, *p* = 0.001: E2 vs E4 *p* = 0.001) with E2 levels being lower than E4. CVS groups were higher than controls (*F*_1, 46_ = 13.74, *p* = 0.001). There was also a sex x genotype interaction (*F*_1, 46_ = 4.30, *p* = 0.019). LDLR/apoE ratios were again dependent on apoE isoform (Fig. 4h. *F*_2, 46_ = 8.29, *p* = 0.001: pairwise comparison: E2 vs E3 *p* = 0.001). Hippocampal, medial prefrontal cortical, and liver tissues did not show significant differences between genotypes or CVS conditions (Supplementary Table 3).

Plasma apoE levels were analyzed, based on evidence for a positive correlation between plasma apoE levels and PTSD symptom severity [37]. E2 mice showed higher levels of plasma apoE compared to E3 and E4 mice (Fig. 4i. *F*_2,52_ = 81.780, *p* < 0.001; E2 vs E3: *p* < 0.001, E2 vs E4: *p* < 0.001). Furthermore, females had higher apoE levels than males (Sex: *F*_1, 52_ = 4.59, *p* = 0.037; Sex x genotype: *F*_2,52_ = 3.70, *p* = 0.031). This sex difference appeared to be driven by E2 females. CVS mice had lower levels compared to controls (Group: *F*_1,52_ = 6.89, *p* = 0.011), which was also driven by the decrease seen in E2 CVS mice (*F*_2,52_ = 6.16, *p* = 0.004).

Within the cortex, as analyzed by ELISA, all CVS groups had lower apoE levels compared to controls (Fig. 4j. *F*_1,58_ = 48.91, *p* < 0.001); sex and genotype were not significant. This is in contrast to what we found using Western blot (Supplementary Table 3) using the same samples. MAP-2 levels were higher in mice exposed to CVS (Fig. 4k. *F*_1, 65_ = 58.57, *p* < 0.001). There was also a genotype x CVS condition interaction (*F*_2,65_ = 3.34, *p* = 0.041). MAP-2 levels were markedly less increased by CVS in E2 than E3 or E4 mice.

### Assessment of cholesterol metabolism

To examine the effect of apoE genotype and the interaction of stress (CVS) on cholesterol metabolism, we assessed cholesterol as well as 7 different sterols (cholestanol, desmosterol, and lathosterol) and oxysterols (24S-hydroxycholesterol, 25-hydroxycholesterol, and 27 hydroxycholesterol) in brain (Fig. 4l–n, Supplementary Table 4). The only one affected by CVS exposure was 7-ketocholesterol (Fig. 4l). Two-way ANOVA revealed a main effect of CVS exposure in which CVS corresponded to higher 7-ketocholesterol cortical levels (*F*_1,26_ = 6.53, *p* = 0.017). There was also an interaction between genotype and CVS condition (*F*_2,26_ = 4.72, *p* = 0.018). E4 mice exposed to CVS had higher 7-ketocholesterol levels than genotype-matched controls (*p* = 0.013, Sidak’s multiple comparisons). This CVS-related difference was absent in E2 mice (*p* = 0.72) and E3 mice (*p* = 0.11).

This genotype x CVS interaction led us to explore 7-ketocholesterol levels in liver, a major organ for cholesterol metabolism, and in the plasma. Exposure to CVS corresponded to higher levels of 7-ketocholesterol in the liver regardless of genotype or sex (Fig. 4m. *F*_1, 36_ = 16.13, *p* < 0.001). This effect of CVS exposure was not seen in plasma; however, E2 mice regardless of sex or CVS exposure showed higher levels of 7-ketcholesterol compared to E3 and E4 mice (Fig. 4n. *F*_2,34_ = 24.42, *p* < 0.001). There were no effects of sex or CVS exposure on plasma levels of 7-ketocholesterol.

### ApoE genotype frequencies in Vietnamese and Cambodian patients

To further understand how apoE genotype may influence PTSD prevalence in a clinical population, we enrolled Vietnamese and Cambodian civilian patients already receiving care at the OHSU IPP. We found that more than half the subjects within the study had been diagnosed with PTSD at some point during their care (Table 1, PTSD- yes (current) or yes (in remission)). Binomial test showed a trend (*p* = 0.0622) that E2 carriers may be more likely diagnosed with PTSD compared to non-E2 carriers based on genotype percentages expected within an older population [38]. Furthermore, E2 carriers had an odds ratio of 1.701 of having a PTSD diagnosis compared to E3/E3 carriers (*p* = 0.45, Fischer’s exact test, two-sided). In contrast, E4 carriers had an odds ratio of 1.137 compared to E3/E3 carriers.

**Table 1:** APOE genotype frequencies and PTSD diagnosis status in Vietnamese and Cambodian patients at the OHSU IPP.

	E2/E3	E2/E4	E3/E3	E3/E4	E4/E4	ALL
Total	18	3	63	15	3	102
Women	13	2	41	10	2	68
Men	5	1	22	5	1	34
Average age	67.11	60.33	62.32	61.33	67	61.08
Vietnamese	14	2	49	13	3	81
Cambodian	4	1	14	2	0	21
PTSD - yes (current)	7	0	22	7	1	37
PTSD - yes (in remission)	5	2	12	2	0	21
PTSD- no	6	1	29	6	2	44
% of genotype with PTSD	66.67	66.67	53.96	60.00	33.33	56.86
% of all with PTSD	20.69	3.49	58.62	15.52	1.72	*E2* + *vs E2-*: *p* = *0.0622 (binomial)*
% Expected in General Older Population	12.5	2.3	60.7	22.1	1.9	

**E2**+: 14 vs 7; 67%; 1.701 odds ratio compared to E3/E3; *p* = 0.45, Fischer’s exact test (two-sided).

**E4**+12 vs 9; 57%; 1.137 odds ratio compared to E3/E3; *p* = 0.99, Fischer’s exact test (two-sided).

## Discussion

This study shows that CVS exposure results in long-term changes, specifically lower home cage activity during the light phase compared to controls, indicative of circadian rhythm disruptions. CVS exposure was also associated with lipid transport and metabolism, including decreased levels of apoE in cortical tissue and increased levels of 7-ketocholesterol in the liver providing evidence of changes related to cholesterol metabolism throughout the body. Furthermore, we found that in Vietnamese and Cambodian patients, E2 carriers had greater odds of having a PTSD diagnosis compared to non-carriers.

Since LDLR and apoE have demonstrated inverse expression levels [39], we analyzed whether the amount of apoE affected the relationship between LDLR and apoE genotype. LDLR normalized to apoE was lowest in adrenal glands of E2 mice. In the cortex, LDLR/apoE ratios were higher in E3 and E4 CVS-exposed mice compared to genotype matched controls, but this CVS difference was absent in E2 mice. Taken with the changes in 7-ketocholesterol, these data suggest that LDLR may be functionally important in the stress response. Consistent with this notion, identification of the *LDLR* SNP, rs5925, showed predictive value of PTSD symptom severity and prevalence 6 months after the 2008 Wenchuan earthquake in adolescents [40].

Human apoE expression in mouse Y1 adrenal cells results in decreases in glucocorticoid secretion and suggests that apoE may modulate cholesterol utilization [41]. Consistent with this, apoE KO mice show increased plasma corticosterone levels after an acute restraint stress compared to wildtype mice [34]. Plasma corticosterone levels were higher in E2 than E3 and E4 mice. Moreover, female E2 mice exposed to CVS showed lower plasma corticosterone levels compared to genotype-matched controls after an anxiety-provoking maze. This is consistent with the excessive negative feedback found in PTSD patients [42, 43]. In contrast to what was seen following an anxiety-provoking maze in our study, E2 male mice exposed to CVS showed higher levels of apoE after an acute restraint stress [16]. This may also be due to the length of time between CVS and the additional stressor.

The increases in GR/apoE in E3 and E4 mice exposed to CVS suggests that more GR may be expressed in response to CVS and that this may be dependent on apoE isoform. LDLR normalized to apoE levels showed a similar difference corresponding to CVS further suggesting a CVS-dependent response. Similar patterns in the adrenal gland emphasize this relationship between GR and apoE is mediated for apoE isoform and that E2 mice may lack the increase in GR relative to apoE needed to signal additional feedback.

This study supports previously noted differences in the adrenal weights of apoE TR mice exposed to CVS [16] that may be a result of decreased feedback inhibition. In the water maze, E2 mice are also susceptible to CVS-related impairments in spatial learning. Specifically, E2 females, but not males, fail to show spatial memory retention. In our previous study, male E2 mice lacked target preference assessed immediately after exposure to fear stress [16], suggesting that this effect might be time-dependent. Consistent with transient effects, male E2 mice decreased in percent baseline body weight during the week of CVS but regained their body weight afterwards. Both E3 and E4 males showed increased body weight after CVS exposure. These data suggest that weight gain seen in PTSD patients may be apoE genotype dependent.

Composition of levels in total (free and unesterified) sterols and oxysterols in young (8 weeks old) male apoE TR mice is similar among E2, E3, and E4 mice but changes depending on genotype by 1 year of age [44]. E2 might then have an effect on cholesterol synthesis and metabolism (perhaps via upregulation of the lathosterol pathway) as well as cholesterol oxidative damage. Taken with our current study findings, the stress response of cholesterol metabolism in young animals may follow a unique pathway compared to what is seen in aging. That is to say, cortical 7-ketocholesterol does not increase in E2 female mice in response to stress as it does with age. Meanwhile, in E3 and E4 female mice, levels of 7-ketocholesterol are only changed in the stress response in young animals.

Plasma analysis of 7-ketocholesterol did not follow the same pattern seen in cortical tissue but E2 mice showed the highest levels regardless of CVS. In the liver, CVS exposure corresponded to increased 7-ketocholesterol regardless of genotype, which may contribute to systemic levels of 7-ketocholesterol. While 7-ketocholesterol can move through the BBB, there are additional mechanisms regulating its degradation and excretion that differ between tissues and circulation.

Differences in E2 mice vs E3 and E4 mice regardless of CVS exposure highlight baseline differences in the apoE TR mice. After CVS, male E2 mice were reported to have higher activity during the light phase compared to E3 and E4 mice and greater anxiety-like behavior [16]. This was not replicated in the current study. Salient differences between the studies may have contributed to these divergent results: (1) This study involved males and females, which were tested at the same time, whereas only males were assessed previously. (2) Mice were pair housed for the entire duration of this study with a littermate except for when CVS mice were singly housed for 5 days during exposure to stressors. Females may be more affected by the effects of social isolation [45, 46], which may play a role in the severe memory impairment seen in females. In this study, we used two common, similar tests of anxiety-like behavior (elevated plus and zero mazes) to assess baseline and post-CVS anxiety-like behavior. Both mazes were used to avoid the potential confound of habituation to the maze itself and showed that E2 mice spent less percent total time in the open areas of both mazes and detected sex differences. CVS did not elicit anxiety-like behavior in this study, but we cannot exclude that this may be due to floor effect (*i.e*. that all mice demonstrated anxiety-like behavior) that limited our ability to detect CVS-dependent differences.

CVS corresponded to increases in cortical MAP-2 levels, perhaps part of a compensatory mechanism. Chronic restraint stress, but not chronic variable stress, resulted in increased dendritic arborization in the hippocampus and amygdala [47]. In addition, in aged mice and nonhuman primates, MAP-2 levels were increased in association with age [48].

In order to underscore the translational relevance of our mouse studies and to better understand the role of *APOE* genotype in PTSD in the context of a civilian non-Caucausian population, we genotyped individuals receiving care at the OHSU IPP. The OHSU IPP has been a long-standing, successful teaching clinic for cross-cultural psychiatry [29]. At the OHSU IPP, PTSD rates were surprisingly high in this population of Southeast Asian refugees after a secondary, more structured interview to assess for PTSD [49]. The cross-cultural nature of treating refugees, especially from countries with vastly different cultures and belief systems, perhaps contributed to this high diagnosis rate during the second interview. As genotype has been previously assessed in PTSD populations, largely in Veteran populations [15–17], we chose to focus on the Vietnamese and Cambodian civilian patients at the IPP. ApoE allele prevalence rates vary widely among different ethnic populations [50]. Even disorders highly correlated to apoE genotype, such as the E4 allele and its associated risk for Alzheimer’s disease and cardiovascular disease are influenced by race and ethnicity [9, 51–53]. Furthermore, the age range of our cohort is especially critical given the altered risk in developing AD after PTSD diagnosis [54–56]. However, research on this intersection has focused so far on the influence of E4 [57].

E2 carriers had a greater odds ratio of having a PTSD diagnosis compared to E3/E3 individuals, while E4 carriers had an odds ratio near 1. It should be noted that due to the nature of the population that the clinic serves, many of these subjects have been patients of the IPP for decades since they relocated to Portland, Oregon. We were not able to directly compare our findings to previous work showing that symptom severity, but not prevalence was worse in E2 carriers compared to noncarriers in a cohort of male combat veterans [16]. While these factors do limit the generalizability of the study, our findings suggest that E2+ genotype may lead to greater incidence of PTSD. As the protective effect of E2 does not appear to be ethnicity-dependent [9], the E2 link with PTSD is perhaps less dependent on ethnicity although certainly more research is needed.

The understanding of trauma as being the emotional response to an event or series of events is relevant to the CVS model and for our human cohort. While PTSD can be linked to a singular event, often it is not. For example, for refugees often the traumatic events compound on top of one another and include emotional and physiological trauma. It is certainly the case that our rodent model has limitations and needs to be part of ongoing studies to better understand the mechanisms that link E2 with PTSD-related symptoms.

In summary, apoE TR mice show isoform-specific responses to CVS, behaviorally, cognitively, and physiologically. E2 mice showed greater memory impairment when exposed to CVS, which was worse in females. Female E2 mice appeared to have a blunted response to CVS compared to that seen in E3 and E4 mice in cortical 7-ketcholesterol levels as well as cortical and adrenal GR/apoE levels. Taken with our human data supporting E2 as a risk allele for PTSD, future studies are warranted to assess how 7-ketocholesterol may respond to extreme stressors and how this could be leveraged for more personalized therapies in stress-related disorders like PTSD.

## Supplementary information

Suppl. Information

Suppl. Table 1

Suppl. Table 2

Suppl. Table 3

Suppl. Table 4

Suppl. Figure 1

Suppl. Figure 2

Suppl. Figure 3

Suppl. Figure 4

## Data Availability

Data will be made available upon reasonable request.
