# Supplementary material for: Apolipoprotein E Isoform-specific changes related to stress and trauma exposure
Source: Transl Psychiatry. 2022 Mar 28;12:125. doi: 10.1038/s41398-022-01848-7 (PMC8960860; doi:10.1038/s41398-022-01848-7)
Supplement: Supplementary file 1 — Suppl. Information [file 41398_2022_1848_MOESM1_ESM.docx]

**Supplementary methods information**

*Experimental design*

Mice were tested among 6 cohorts with sex and genotype distributed throughout. Group sizes of each sex within genotypes for CVS exposure were as follows: Control Females: E2 = 11, E3 = 12, E4 = 12; Control Males: E2 = 12, E3 = 12, E4 = 12; CVS Females: E2 = 12, E3 = 14, E4 = 12; CVS Males: E2 = 16, E3 = 14, E4 = 12. Group sizes were based on effect sizes seen in previous experiments. Control and CVS mice were tested in separate cohorts to avoid potential confounds. Mice were stratified by age to be in either the Control or CVS groups before the experiment began. The same experimenter handled and tested all mice while blind to the genotype and sex. One mouse was treated for malocclusion throughout the testing and two were euthanized for health reasons unrelated to CVS.

*CVS*

Stressors included social deprivation (*i.e*., single housing), 30° cage tilt for 3 hrs, wet home cage for 3 hrs, overnight food deprivation, 3-min cold swim (10-12°C), and 15-min restraint. Mice were exposed to 2 unique stressors at random times throughout each day.

### Behavioral testing

Mice were pair-housed at the start of the experiment. Body weights were recorded weekly. During Week 2, control mice remained in their home cages while CVS mice were singly-housed and exposed to stressors. Home cage activity was recorded with noninvasive home cage monitors (MLog, Biobserve, Germany) as described^1^. Activity was analyzed as 30-min averages using R (R Foundation for Statistical Computing, Vienna, Austria). Exposure to CVS took place over the course of 5 days as described^2^ (details in **Supplementary Fig. 1**).

Measures of anxiety were assessed in the elevated plus maze and elevated zero maze as described^3^. Light levels were 80-100 lux.

The water maze was used to assess hippocampal-dependent spatial memory as described^2^. The water maze consisted of a 140 cm diameter pool filled with water (21°C ± 2°C) surrounded by large spatial cues. A small circular platform (10 cm in diameter) was placed 1 cm below the water surface. White chalk was added to the water to hide the platform location. Mice were given 2 trials per session with 2 session daily and were first trained to locate a visible flag to learn to escape from the maze by stepping onto the hidden, submerged platform. Each trial was 60 s; if the mouse did not locate the platform, it was led to the platform. The platform location was changed after each session to avoid procedural learning. The morning after the last session hidden platform training, spatial memory retention was assessed in a probe trial (no platform). Swim speed, latency to the target, cumulative distance to the target, time spent in each quadrant, and time spent in the periphery were analyzed using Ethovision 7.1 XT software (Noldus, Netherlands).

### Plasma and tissue collection and preparation

Immediately after elevated zero maze testing, blood was collected from the mandibular vein in unanesthetized mice with 0.5M EDTA and centrifuged at 40,00 *g* for 10 min at 4°C to collect plasma. Three hours after blood collection, mice were anesthetized with a lethal ketamine cocktail and perfused with 1x PBS. Brain regions, including the frontal cortex, medial prefrontal cortex, and hippocampus^4,5^, along with the adrenal gland, liver, and kidney were dissected and flash frozen using liquid nitrogen and kept at -80°C until further use. Tissues were homogenized in a lysis buffer consisting of 1M Tris-Cl, 6M NaCl, 10% SDS, and 0.5M EDTA, 1% Triton-X, and protease inhibitor, (Roche, Sigma Aldrich, catalog #11836170001, St. Louis, MO). Total protein amounts were determined by BCA protein assay kit (Pierce, Thermo Scientific, catalog #23225, Waltham, MA).

### Radioimmune assay for corticosterone

Plasma corticosterone was analyzed using a radioimmune assay kit (MP Biomedicals, catalog #7120102, Irvine, CA). Intra-assay coefficient of variation was 10% and the inter-assay coefficient of variation was 7%.

### Protein measurements

Pilot studies were performed to ensure that target protein levels for Western blot analysis were within the linear range of the fluorescence signal; 15 mg of total protein for each sample was loaded for all tissues except for the mPFC, for which 10 mg of total protein per sample was loaded. Samples were run in duplicate and all groups were included on each individual blot. As housekeeping genes can be altered by experimental conditions ^6,7^, total protein was used for normalization and measured using the Bio-Rad Total Protein Stain-Free gel system imaged on the Azure c600 (San Francisco, CA) ( See **Supplementary Fig. 2**).

Tissue homogenates were prepared with a Laemmli sample buffer and β-mercaptoethanol mixture and run on SDS-PAGE gels (Bio-Rad, 4-15% TGX Total Protein Stain-Free gels, 26 wells, Hercules, CA) for 42 min at 200 V. Proteins were then transferred to PVD membranes using a wet transfer system with 1x Tris/Glycine with 20% methanol buffer solution for 30 min at 100 V.

Blots were rinsed and blocked with Azure fluorescent blocking buffer. Primary antibody solutions (Millipore Calbiochem catalog #178479 goat anti-apoE 1:4000 at 32-34 kD, Burlington, MA; R&D catalog #AF2255 goat anti-LDLR 1:1000 at 135 kD, Minneapolis, MN; and Cell-Signaling, catalog #12041S rabbit anti-GR 1:2000 at 91, 94 kD, Danvers, MA) were then used for overnight incubation at 4ºC. Blots were rinsed and incubated in secondary antibody solutions (LiCor IRDye 680RD Donkey anti-rabbit 1:10,000, LiCor IRDye 800CW donkey anti-goat 1:10,000, Lincoln, NE) before being rinsed again. Images were acquired using an Azure c600 at the corresponding fluorescent wavelength. Target proteins and total protein were analyzed using AzureSpot software. All target proteins were normalized to the total protein for each sample.

ApoE (Millipore Sigma, RAB0613-1KT, Burlington, MA) and MAP-2 (MyBioSource, MBS725632, San Diego, CA) were also analyzed using ELISAs and spectrophotometrical measures at 450 nm using a SpectraMax iD5 microplate reader (Molecular Devices, San Jose, CA). Standards were run in duplicate and samples run in singlets. For the apoE ELISA, the intra-assay CV is <10% and the sensitivity is 1.5 ng/mL. The inter-assay CV for the MAP-2 ELISA is < 9% and the sensitivity is 0.1 ng/mL. Graphpad Prism software (Prism, La Jolla, CA, USA) was used to calculate the best-fit curve for the standards, and sample results were interpolated based on the standard curve.

### Cholesterol & related metabolite analyses

Cortical tissues (30 mg) were homogenized in 1x PBS using homogenization beads. Following saponification, samples were extracted with hexane and derived with N,N-dimethylglycine (DMG)^8,9^. Total 7-ketocholesterol, 24S-hydroxycholesterol, 25-hydroxycholesterol and 27-hydroxycholesterol were determined by LC-MS/MS.

Samples (75 µL) were spiked with 5 µL of internal standard mixture containing 7-ketocholesterol-d7 1ng/µl, 25-hydroxycholesterol-d6 2 ng/µl and 27-hydroxycholesterol-d6 20 ng/µl in methanol. Standards were prepared in homogenization buffer. Saponification was accomplished by diluting sample with 2 mL of ethanol followed by 0.120 mL of 33% KOH. Samples were vortexed then heated at 37ºC for 1 hr. After saponification, each sample was diluted with 2 mL of water and extracted twice with 4 mL of hexane. The combined hexane extracts were dried under vacuum. After drying the tubes were rinsed with 0.4 mL of hexane and dried again. The dried sample was treated with 25 µl mixture of DMG at 0.5M and 4-(N,N-dimethylamino)pyridine at 1M in chloroform and 25 µl 1-ethyl-3-(3-dimethylaminopropyl)carbodiimide at 1M chloroform, then heated at 45°C. After 1 hr, 50 µl of methanol was added to deactivate the excess derivatizing agent. Samples were dried down in speed vacuum, suspended in 100 µl of methanol, vortexed, centrifuged, and filtered prior to analysis of 5 µl injection with LC-MS/MS.

DMG Derivatives were analyzed using a 4000 Q-TRAP hybrid/triple quadrupole linear ion trap mass spectrometer (SCIEX, Framingham, MA) with electrospray ionization (ESI) in positive mode. The mass spectrometer was interfaced to a Shimadzu (Columbia, MD) SIL-20AC XR auto-sampler followed by 2 LC-20AD XR LC pumps. The instrument was operated with the following settings: source voltage 4000 kV, GS1 40, GS2 30, CUR 40, TEM 500 and CAD gas medium. Compounds transitions were quantified with multiple reaction monitoring (MRM) with peak retention times as described in **Supplementary Table 1**.

Separation was achieved using an ACE Excel 3 µm C18-PFP 100x2.1mm (ACE, part # EXL-1110-1002U) column kept at 18°C using a Shimadzu CTO-20AC column oven. The gradient mobile phase was delivered at a flow rate of 0.4 mL/min between 0-6.5 min, 0.8 mL/min between 6.6-10 min and 0.4 mL/min between 10.1-12 min, and consisted of two solvents, A: 0.1% formic acid, 2mM ammonium acetate in water:methanol at 95:5 v/v, B: 0.1% formic acid, 2 mM ammonium acetate in methanol:acetonitrile at 10:90 v/v. The initial concentration of solvent B was 55% followed by a linear increase to 70% B in 3 min, then to 100% B in 2.5 min, held for 4 min, decreased back to starting 55% B over 0.1 min, and then held for 2.5 min. Data were acquired using Analyst 1.6.2 and analyzed with Multiquant 3.0.3 software (SCIEX, Framingham, MA). Sample values were calculated from standard curves generated from the peak area ratio of the analyte to internal standard versus the analyte concentration that was fit to a linear equation with 1/x weighting. Analytical measurement range was 5-1,000 ng/mL homogenate.

After determining that 7-ketocholesterol was of primary interest from the cortical tissue results, liver tissue samples, homogenized similarly in 1x PBS, and plasma samples were assayed for free 7-ketocholesterol levels, similarly to the above method but without the saponification step due to matrix interference determined in pilot liver and plasma samples. Cholesterol was measure in hippocampal homogenates using previously described methods^10^. Cholestanol, desmosterol, and lanosterol were measured in hippocampal homogenates using GC-MS after a chloroform:methanol (2:1) extraction and saponification with ethanol/KOH. Due to technical limitations, sterols were assessed only in male hippocampal tissues and oxysterols were only analyzed in female cortical tissues.

Hippocampal cholesterol analyses

For hippocampal cholesterol analyses, lipids were extracted from hippocampal homogenates by chloroform:methanol (2:1) extraction. Internal standard was added and the samples were dried and saponified with alcoholic KOH and extracted into hexane. Samples were derivatized with N,O-Bis(trimethylsilyl)trifluoroacetamide (BSTFA) (Thermo-Scientific, Rockford, IL) and cholesterol concentration was measured by capillary column gas chromatography on an Agilent (Santa Clara, CA) gas chromatograph (Model 6890N) with a ZB1701 column (30 m, 0.25 mm ID, 0.25 μm film; Phenomenex, Torrance, CA) and a FID detector. An internal standard (epicoprostanol; Sigma, St. Louis, MO) and an authentic cholesterol standard (Steraloids, Newport, RI) were used for calibration.

Sterol panel in hippocampal tissue

Gas chromatography-mass spectrometry (GC-MS) was used to measure cholestanol, desmosterol, and lanosterol. An internal standard (epicoprostanol) was added to hippocampal homogenates and sterols were extracted by chloroform:methanol (2:1) extraction. Extracts were dried and sterols were saponified by the addition of ethanol/KOH, incubated at 37°C for one hour and the aqueous phase was extracted twice with hexane. Concentrations of the trimethylsilyl ether derivatives of sterols were measured using GC performed with a ZB1701 column (Phenomenex, Torrance, CA) coupled to a mass spectrometer (Agilent GC 6890N and MS 5975; Santa Clara, CA). Mass spectra were collected in selected ion mode with m/z= 355 and 370 ions monitored for epicoprostanol internal standard (quantifying and qualifying ions respectively), m/z = 393.2 and 498.2 ions for lanosterol, m/z = 343.3 and 441.5 ions for desmosterol, and m/z = 458.5 and 255.3 ions for lathosterol. Calibrants were generated using authentic standards (cholestanol, desmosterol, and lathosterol from Avanti Polar Lipids, Alabaster, AL). Analyte concentrations were calculated across the range 0.04–3.2 mg/dL using calibration curves generated by performing a least-squares linear regression for peak area ratios plotted against specified calibrant concentration. The lower limit of quantification was determined as the lowest spiked concentration in matrix for which the signal-to-noise ratio was ≥ 5. The between-run precision of the assay was determined to be < 20% relative standard deviation.

*Subject enrollment, saliva sample collection and genotyping*

Subjects of Cambodian or Vietnamese background were recruited at the OHSU IPP. Accredited counselors provided translation for the study as most of the subjects do not speak English. Upon enrolling in the study, subjects signed consent forms, which were made available in Vietnamese or Khmer. Saliva samples were collected for apoE genotyping, coded with a study ID, and frozen in a secure -20°C freezer. Staff at the Oregon Clinical and Translational Research Institute determined apoE genotype. Information regarding sex, age, ethnicity, PTSD diagnosis (none, current, in remission, etc.), primary PTSD symptoms, PTSD severity at time of diagnosis, presence of other health conditions (specifically, diabetes, cardiovascular disease, hypertension, sleep problems, substance use disorders, and smoking status), presence of comorbid psychiatric disorders, and current medication were also retrieved from patient health records. Since most subjects identified as Vietnamese, we did not analyze Vietnamese and Cambodian-identifying groups separately. All documents and protocols were approved of by the OHSU Institutional Review Board (IRB Protocol #16968).

### Statistical analyses

Behavioral and biochemical data are reported as mean ± standard error of the mean and were analyzed using SPSS v25 (IBM, Armonk, NY, USA). GraphPad software v.8.2.0 was used to visualize findings (Prism, La Jolla, CA, USA). Multi-factorial analyses of variance were used, including genotype, sex, and stress exposure as factors with Sidak’s correction for multiple comparisons. For water maze training data, mean swim speed was included as a covariate. For Western blot data, blot was included as a covariate for analyses to account for differences in staining. Estimated marginal means were used for pairwise comparisons. Repeated measures were used when necessary and when Mauchly’s test of sphericity was significant, Greenhouse-Geisser corrections were applied.

Due to the low expected frequencies of E2 and E4 in the patient cohort, genotypes were assessed by grouping all E2+ (E2/E3, E2/E4) were compared to E2- and subsequently all E4+ (E3/E4, E2/E4, and E4/E4) to E4- subjects. Binomial tests were used. Significance was set at *p ≤* 0.05.

1 Weiss, J. B., Weber, S. J., Torres, E. R. S., Marzulla, T. & Raber, J. Genetic inhibition of Anaplastic Lymphoma Kinase rescues cognitive impairments in Neurofibromatosis 1 mutant mice. *Behav Brain Res* **321**, 148-156, doi:10.1016/j.bbr.2017.01.003 (2017).

2 Johnson, L. A. *et al.* ApoE2 Exaggerates PTSD-Related Behavioral, Cognitive, and Neuroendocrine Alterations. *Neuropsychopharmacology* **40**, 2443-2453, doi:10.1038/npp.2015.95 (2015).

3 Siegel, J. A., Haley, G. E. & Raber, J. Apolipoprotein E isoform-dependent effects on anxiety and cognition in female TR mice. *Neurobiol Aging* **33**, 345-358, doi:10.1016/j.neurobiolaging.2010.03.002 (2012).

4 Pflibsen, L. *et al.* Executive function deficits and glutamatergic protein alterations in a progressive 1-methyl-4-phenyl-1,2,3,6-tetrahydropyridine mouse model of Parkinson's disease. *Journal of neuroscience research* **93**, 1849-1864, doi:10.1002/jnr.23638 (2015).

5 Raber, J. *et al.* Combined Effects of Three High-Energy Charged Particle Beams Important for Space Flight on Brain, Behavioral and Cognitive Endpoints in B6D2F1 Female and Male Mice. *Frontiers in physiology* **10**, 179, doi:10.3389/fphys.2019.00179 (2019).

6 Torres, E. R. S. *et al.* Effects of Sub-Chronic MPTP Exposure on Behavioral and Cognitive Performance and the Microbiome of Wild-Type and mGlu8 Knockout Female and Male Mice. *Frontiers in Behavioral Neuroscience* **12**, doi:10.3389/fnbeh.2018.00140 (2018).

7 Eaton, S. L. *et al.* Total Protein Analysis as a Reliable Loading Control for Quantitative Fluorescent Western Blotting. *PLOS ONE* **8**, e72457, doi:10.1371/journal.pone.0072457 (2013).

8 Jiang, X., Ory, D. S. & Han, X. Characterization of oxysterols by electrospray ionization tandem mass spectrometry after one-step derivatization with dimethylglycine. *Rapid Commun Mass Spectrom* **21**, 141-152, doi:10.1002/rcm.2820 (2007).

9 Pataj, Z., Liebisch, G., Schmitz, G. & Matysik, S. Quantification of oxysterols in human plasma and red blood cells by liquid chromatography high-resolution tandem mass spectrometry. *J Chromatogr A* **1439**, 82-88, doi:10.1016/j.chroma.2015.11.015 (2016).

10 Johnson, L. A. *et al.* Apolipoprotein E-low density lipoprotein receptor interaction affects spatial memory retention and brain ApoE levels in an isoform-dependent manner. *Neurobiol Dis* **64**, 150-162, doi:10.1016/j.nbd.2013.12.016 (2014).
