# Supplementary material for: Apolipoprotein E Isoform-specific changes related to stress and trauma exposure
Source: Transl Psychiatry. 2022 Mar 28;12:125. doi: 10.1038/s41398-022-01848-7 (PMC8960860; doi:10.1038/s41398-022-01848-7)
Supplement: Supplementary file 2 — Suppl. Table 1 [file 41398_2022_1848_MOESM2_ESM.docx]

**Supplementary Table 1.** Oxysterol multiple reaction monitoring (MRM) transitions. Quantifying (quan) and qualifying (qual) ion information. *Note that 25OH-C-d6-DMG quan internal standard was used for quantification of 24SOH-C-DMG quan.

| **Q1 mass** | **Q3 mass** | **Retention time (min)** | **Analyte** | **DP (V)** | **EP (V)** | **CE (V)** | **CXP (V)** |
| --- | --- | --- | --- | --- | --- | --- | --- |
| **488.4** | 385.4 | 4.4 | 24SOH-C-DMG quan* | 81 | 10 | 23 | 12 |
| **488.4** | 367.4 | 4.4 | 24SOH-C-DMG qual* | 81 | 10 | 29 | 12 |
| **488.4** | 104.1 | 4.7 | 27OH-C-DMG quan | 101 | 10 | 39 | 20 |
| **488.4** | 58.2 | 4.7 | 27OH-C-DMG qual | 101 | 10 | 91 | 10 |
| **494.4** | 58.2 | 4.7 | 27OH-C-d6-DMG quan | 101 | 10 | 91 | 10 |
| **488.4** | 367.4 | 4.0 | 25OH-C-DMG quan | 81 | 10 | 29 | 12 |
|  |  |  |  |  |  |  |  |
| **488.4** | 385.4 | 4.0 | 25OH-C-DMG qual | 81 | 10 | 23 | 12 |
| **494.4** | 373.4 | 4.0 | 25OH-C-d6-DMG quan | 81 | 10 | 29 | 12 |
| **486.4** | 58.2 | 7.2 | 7-KC-DMG quan | 76 | 10 | 87 | 10 |
| **486.4** | 104.1 | 7.2 | 7-KC-DMG qual | 76 | 10 | 37 | 18 |
| **493.4** | 390.2 | 7.2 | 7-KC-d7-DMG quan | 76 | 10 | 27 | 12 |
