# Supplementary material for: Apolipoprotein E Isoform-specific changes related to stress and trauma exposure
Source: Transl Psychiatry. 2022 Mar 28;12:125. doi: 10.1038/s41398-022-01848-7 (PMC8960860; doi:10.1038/s41398-022-01848-7)
Supplement: Supplementary file 3 — Suppl. Table 2 [file 41398_2022_1848_MOESM3_ESM.pdf]

**Supplementary Table 2. Additional statistical analyses of percent time spent in each quadrant in during probe trials.**

**Probe Trial 1**

| Group | Model | Effect | df | Error | *F* | *p* |
|---|---|---|---|---|---|---|
| *Female E2 Controls* | RM ANOVA | Within-subject: Quadrant | 1.939 | 19.386 | 6.766 | **0.006** |
| | Mauchly's test: p = 0.049 | Pairwise comparisons | | | | |
| | | Target Quadrant vs Right Quadrant : | | | | **0.017** |
| | | Target Quadrant vs Left Quadrant : | | | | 0.632 |
| | | Target Quadrant vs Opposite Quadrant : | | | | 0.53 |
| *Female E2 CVS* | RM ANOVA | Within-subject: Quadrant | 1.699 | 18.69 | 1.167 | 0.325 |
| | Mauchly's test: p = 0.043 | Pairwise comparisons | | | | |
| | | Target Quadrant vs Right Quadrant : | | | | 0.221 |
| | | Target Quadrant vs Left Quadrant : | | | | 0.112 |
| | | Target Quadrant vs Opposite Quadrant : | | | | 0.997 |
| *Female E3 Controls* | RM ANOVA | Within-subject: Quadrant | 3 | 33 | 18.146 | **< 0.001** |
| | Mauchly's test: p = 0.192 | Pairwise comparisons | | | | |
| | | Target Quadrant vs Right Quadrant : | | | | 0.145 |
| | | Target Quadrant vs Left Quadrant : | | | | **< 0.001** |
| | | Target Quadrant vs Opposite Quadrant : | | | | **0.001** |
| *Female E3 CVS* | RM ANOVA | Within-subject: Quadrant | 3 | 39 | 7.777 | **< 0.001** |
| | Mauchly's test: p = 0.054 | Pairwise comparisons | | | | |
| | | Target Quadrant vs Right Quadrant : | | | | 0.121 |
| | | Target Quadrant vs Left Quadrant : | | | | **0.008** |
| | | Target Quadrant vs Opposite Quadrant : | | | | **0.001** |
| *Female E4 Controls* | RM ANOVA | Within-subject: Quadrant | 3 | 33 | 4.311 | **0.011** |
| | Mauchly's test: p = 0.318 | Pairwise comparisons | | | | |
| | | Target Quadrant vs Right Quadrant : | | | | 0.955 |
| | | Target Quadrant vs Left Quadrant : | | | | **0.011** |
| | | Target Quadrant vs Opposite Quadrant : | | | | 0.056 |
| *Female E4 CVS* | RM ANOVA | Within-subject: Quadrant | 3 | 30 | 4.863 | **0.007** |
| | Mauchly's test: p = 0.310 | Pairwise comparisons | | | | |
| | | Target Quadrant vs Right Quadrant : | | | | 0.169 |
| | | Target Quadrant vs Left Quadrant : | | | | 0.089 |
| | | Target Quadrant vs Opposite Quadrant : | | | | 0.17 |
| *Male E2 Controls* | RM ANOVA | Within-subject: Quadrant | 3 | 30 | 9.408 | **< 0.001** |
| | Mauchly's test: p = 0.259 | Pairwise comparisons | | | | |
| | | Target Quadrant vs Right Quadrant : | | | | **0.001** |
| | | Target Quadrant vs Left Quadrant : | | | | 0.622 |
| | | Target Quadrant vs Opposite Quadrant : | | | | **0.013** |
| *Male E2 CVS* | RM ANOVA | Within-subject: Quadrant | 3 | 45 | 5.27 | **0.003** |
| | Mauchly's test: p = 0.096 | Pairwise comparisons | | | | |
| | | Target Quadrant vs Right Quadrant : | | | | 0.854 |
| | | Target Quadrant vs Left Quadrant : | | | | 0.053 |
| | | Target Quadrant vs Opposite Quadrant : | | | | 0.062 |
| *Male E3 Controls* | RM ANOVA | Within-subject: Quadrant | 3 | 33 | 24.361 | **< 0.001** |
| | Mauchly's test: p = 0.077 | Pairwise comparisons | | | | |
| | | Target Quadrant vs Right Quadrant : | | | | **0.004** |
| | | Target Quadrant vs Left Quadrant : | | | | **< 0.001** |
| | | Target Quadrant vs Opposite Quadrant : | | | | **0.001** |
| *Male E3 CVS* | RM ANOVA | Within-subject: Quadrant | 3 | 39 | 12.642 | **< 0.001** |
| | Mauchly's test: p = 0.272 | Pairwise comparisons | | | | |
| | | Target Quadrant vs Right Quadrant : | | | | **0.002** |
| | | Target Quadrant vs Left Quadrant : | | | | **0.008** |
| | | Target Quadrant vs Opposite Quadrant : | | | | **0.005** |
| *Male E4 Controls* | RM ANOVA | Within-subject: Quadrant | 1.705 | 18.756 | 4.597 | **0.009** |
| | Mauchly's test: p = 0.019 | Pairwise comparisons | | | | |
| | | Target Quadrant vs Right Quadrant : | | | | 0.362 |
| | | Target Quadrant vs Left Quadrant : | | | | **0.001** |
| | | Target Quadrant vs Opposite Quadrant : | | | | 0.243 |
| *Male E4 CVS* | RM ANOVA | Within-subject: Quadrant | 3 | 33 | 17.342 | **< 0.001** |
| | Mauchly's test: p = 0.386 | Pairwise comparisons | | | | |
| | | Target Quadrant vs Right Quadrant : | | | | **0.01** |
| | | Target Quadrant vs Left Quadrant : | | | | **< 0.001** |
| | | Target Quadrant vs Opposite Quadrant : | | | | **0.003** |

| Probe Trial 2 | | | | | |
|---|---|---|---|---|---|
| **Model** | **Effect** | **df** | **Error** | ***F*** | ***p*** |
| RM ANOVA | Within-subject: Quadrant | 1.473 | 14.731 | 3.829 | 0.057 |
| Mauchly's test: p = 0.001 | Pairwise comparisons | | | | |
| | Target Quadrant vs Right Quadrant : | | | | 0.186 |
| | Target Quadrant vs Left Quadrant : | | | | 0.999 |
| | Target Quadrant vs Opposite Quadrant : | | | | 0.743 |
| RM ANOVA | Within-subject: Quadrant | | | | |
| Mauchly's test: p = 0.114 | Pairwise comparisons | | | | |
| | Target Quadrant vs Right Quadrant : | | | | 0.861 |
| | Target Quadrant vs Left Quadrant : | | | | 0.984 |
| | Target Quadrant vs Opposite Quadrant : | | | | 0.761 |
| RM ANOVA | Within-subject: Quadrant | 3 | 33 | 7.685 | **< 0.001** |
| Mauchly's test: p = 0.296 | Pairwise comparisons | | | | |
| | Target Quadrant vs Right Quadrant : | | | | **0.048** |
| | Target Quadrant vs Left Quadrant : | | | | 0.988 |
| | Target Quadrant vs Opposite Quadrant : | | | | **0.019** |
| RM ANOVA | Within-subject: Quadrant | 3 | 39 | 1.883 | 0.148 |
| Mauchly's test: p = 0.664 | Pairwise comparisons | | | | |
| | Target Quadrant vs Right Quadrant : | | | | 0.483 |
| | Target Quadrant vs Left Quadrant : | | | | 0.991 |
| | Target Quadrant vs Opposite Quadrant : | | | | 0.999 |
| RM ANOVA | Within-subject: Quadrant | 3 | 33 | 1.539 | 0.223 |
| Mauchly's test: p = 0.049 | Pairwise comparisons | | | | |
| | Target Quadrant vs Right Quadrant : | | | | 0..358 |
| | Target Quadrant vs Left Quadrant : | | | | 1 |
| | Target Quadrant vs Opposite Quadrant : | | | | 0.992 |
| RM ANOVA | Within-subject: Quadrant | 3 | 30 | 3.718 | **0.022** |
| Mauchly's test: p = 0.063 | Pairwise comparisons | | | | |
| | Target Quadrant vs Right Quadrant : | | | | **0.035** |
| | Target Quadrant vs Left Quadrant : | | | | 0.999 |
| | Target Quadrant vs Opposite Quadrant : | | | | 0.974 |
| RM ANOVA | Within-subject: Quadrant | 3 | 30 | 6.962 | **0.001** |
| Mauchly's test: p = 0.158 | Pairwise comparisons | | | | |
| | Target Quadrant vs Right Quadrant : | | | | 0.113 |
| | Target Quadrant vs Left Quadrant : | | | | 0.248 |
| | Target Quadrant vs Opposite Quadrant : | | | | 0.932 |
| RM ANOVA | Within-subject: Quadrant | 3 | 45 | 3.615 | **0.02** |
| Mauchly's test: p = 0.106 | Pairwise comparisons | | | | |
| | Target Quadrant vs Right Quadrant : | | | | 0.807 |
| | Target Quadrant vs Left Quadrant : | | | | 1 |
| | Target Quadrant vs Opposite Quadrant : | | | | **0.036** |
| RM ANOVA | Within-subject: Quadrant | 3 | 33 | 12.869 | **< 0.001** |
| Mauchly's test: p = 0.307 | Pairwise comparisons | | | | |
| | Target Quadrant vs Right Quadrant : | | | | **0.003** |
| | Target Quadrant vs Left Quadrant : | | | | 0.518 |
| | Target Quadrant vs Opposite Quadrant : | | | | 0.796 |
| RM ANOVA | Within-subject: Quadrant | 1.873 | 24.351 | 6.939 | **0.005** |
| Mauchly's test: p = 0.011 | Pairwise comparisons | | | | |
| | Target Quadrant vs Right Quadrant : | | | | 0.513 |
| | Target Quadrant vs Left Quadrant : | | | | **0.039** |
| | Target Quadrant vs Opposite Quadrant : | | | | 0.679 |
| RM ANOVA | Within-subject: Quadrant | 3 | 33 | 8.168 | **< 0.001** |
| Mauchly's test: p = 0.102 | Pairwise comparisons | | | | |
| | Target Quadrant vs Right Quadrant : | | | | **0.007** |
| | Target Quadrant vs Left Quadrant : | | | | 0.26 |
| | Target Quadrant vs Opposite Quadrant : | | | | 0.889 |
| RM ANOVA | Within-subject: Quadrant | 3 | 33 | 2.844 | 0.053 |
| Mauchly's test: p = 0.346 | Pairwise comparisons | | | | |
| | Target Quadrant vs Right Quadrant : | | | | 0.57 |
| | Target Quadrant vs Left Quadrant : | | | | 0.416 |
| | Target Quadrant vs Opposite Quadrant : | | | | 0.982 |

| Probe Trial 3 | | | | | |
|---|---|---|---|---|---|
| **Model** | **Effect** | **df** | **Error** | ***F*** | ***p*** |
| RM ANOVA | Within-subject: Quadrant | 3 | 30 | 5.274 | **0.045** |
| Mauchly's test: p = 0.406 | Pairwise comparisons | | | | |
| | Target Quadrant vs Right Quadrant : | | | | 0.997 |
| | Target Quadrant vs Left Quadrant : | | | | 0.733 |
| | Target Quadrant vs Opposite Quadrant : | | | | 0.171 |
| RM ANOVA | Within-subject: Quadrant | 3 | 33 | 6.15 | **0.002** |
| Mauchly's test: p = 0.152 | Pairwise comparisons | | | | |
| | Target Quadrant vs Right Quadrant : | | | | 1 |
| | Target Quadrant vs Left Quadrant : | | | | 0.949 |
| | Target Quadrant vs Opposite Quadrant : | | | | **0.002** |
| RM ANOVA | Within-subject: Quadrant | 1.669 | 18.357 | 3.262 | 0.069 |
| Mauchly's test: p = 0.022 | Pairwise comparisons | | | | |
| | Target Quadrant vs Right Quadrant : | | | | 1 |
| | Target Quadrant vs Left Quadrant : | | | | 1 |
| | Target Quadrant vs Opposite Quadrant : | | | | 0.52 |
| RM ANOVA | Within-subject: Quadrant | 3 | 39 | 6.272 | **0.001** |
| Mauchly's test: p = 0.074 | Pairwise comparisons | | | | |
| | Target Quadrant vs Right Quadrant : | | | | 0.991 |
| | Target Quadrant vs Left Quadrant : | | | | 0.884 |
| | Target Quadrant vs Opposite Quadrant : | | | | 0.084 |
| RM ANOVA | Within-subject: Quadrant | 3 | 33 | 0.769 | 0.519 |
| Mauchly's test: p = 0.166 | Pairwise comparisons | | | | |
| | Target Quadrant vs Right Quadrant : | | | | 1 |
| | Target Quadrant vs Left Quadrant : | | | | 1 |
| | Target Quadrant vs Opposite Quadrant : | | | | 0.916 |
| RM ANOVA | Within-subject: Quadrant | 3 | 30 | 3.701 | **0.022** |
| Mauchly's test: p = 0.468 | Pairwise comparisons | | | | |
| | Target Quadrant vs Right Quadrant : | | | | 0.901 |
| | Target Quadrant vs Left Quadrant : | | | | 0.245 |
| | Target Quadrant vs Opposite Quadrant : | | | | 0.066 |
| RM ANOVA | Within-subject: Quadrant | 3 | 30 | 2.474 | 0.081 |
| Mauchly's test: p = 0.739 | Pairwise comparisons | | | | |
| | Target Quadrant vs Right Quadrant : | | | | 0.99 |
| | Target Quadrant vs Left Quadrant : | | | | 0.811 |
| | Target Quadrant vs Opposite Quadrant : | | | | 0.625 |
| RM ANOVA | Within-subject: Quadrant | 2 | 45 | 1.457 | 0.239 |
| Mauchly's test: p = 0.299 | Pairwise comparisons | | | | |
| | Target Quadrant vs Right Quadrant : | | | | 1 |
| | Target Quadrant vs Left Quadrant : | | | | 0.901 |
| | Target Quadrant vs Opposite Quadrant : | | | | 0.357 |
| RM ANOVA | Within-subject: Quadrant | 3 | 33 | 2.707 | 0.061 |
| Mauchly's test: p = 0.252 | Pairwise comparisons | | | | |
| | Target Quadrant vs Right Quadrant : | | | | 0.196 |
| | Target Quadrant vs Left Quadrant : | | | | 0.813 |
| | Target Quadrant vs Opposite Quadrant : | | | | 0.966 |
| RM ANOVA | Within-subject: Quadrant | 3 | 39 | 5.355 | **0.003** |
| Mauchly's test: p = 0.054 | Pairwise comparisons | | | | |
| | Target Quadrant vs Right Quadrant : | | | | 0.847 |
| | Target Quadrant vs Left Quadrant : | | | | 1 |
| | Target Quadrant vs Opposite Quadrant : | | | | 0.103 |
| RM ANOVA | Within-subject: Quadrant | 3 | 33 | 15.771 | **< 0.001** |
| Mauchly's test: p = 0.276 | Pairwise comparisons | | | | |
| | Target Quadrant vs Right Quadrant : | | | | 1 |
| | Target Quadrant vs Left Quadrant : | | | | 0.984 |
| | Target Quadrant vs Opposite Quadrant : | | | | **0.004** |
| RM ANOVA | Within-subject: Quadrant | 1.78 | 19.581 | 3.174 | 0.069 |
| Mauchly's test: p < 0.001 | Pairwise comparisons | | | | |
| | Target Quadrant vs Right Quadrant : | | | | 0.426 |
| | Target Quadrant vs Left Quadrant : | | | | 0.878 |
| | Target Quadrant vs Opposite Quadrant : | | | | 0.051 |
