# Supplementary material for: Apolipoprotein E Isoform-specific changes related to stress and trauma exposure
Source: Transl Psychiatry. 2022 Mar 28;12:125. doi: 10.1038/s41398-022-01848-7 (PMC8960860; doi:10.1038/s41398-022-01848-7)
Supplement: Supplementary file 4 — Suppl. Table 3 [file 41398_2022_1848_MOESM4_ESM.docx]

| **Tissue** | **Measure** | **E2** | | **E3** | | **E4** | | ***p*** |
| --- | --- | --- | --- | --- | --- | --- | --- | --- |
|  |  | **Control** | **CVS** | **Control** | **CVS** | **Control** | **CVS** |  |
| **Cortex** | *apoE/Tot Prot* | 1.26 ± 0.27 | 1.15 ± 0.23 | 1.18 ± 0.28 | 1.01 ± 0.22 | 1.04 ± 0.23 | 0.81 ± 0.16 | n.s. |
|  | *LDLR/Tot Prot* | 0.056 ± 0.013 | 0.052 ± 0.011 | 0.050 ± 0.011 | 0.062 ± 0.016 | 0.051 ± 0.013 | 0.061 ± 0.012 | n.s. |
|  | *GR/Tot Prot* | 0.74 ± 0.11 | 0.71 ± 0.093 | 0.79 ± 0.11 | 1.08 ± 0.15 | 0.82 ± 0.11 | 0.98 ± 0.15 | n.s. |
| **Adrenal Gland** | *LDLR/Tot Prot* | 1.125 ± 0.12 | 1.21 ± 0.16 | 1.00 ± 0.055 | 1.094 ± 0.12 | 1.14 ± 0.13 | 0.98 ± 0.090 | n.s. |
| **Hippocampus** | *apoE/Tot Prot* | 1.31 ± 0.093 | 1.33 ± 0.10 | 1.24 ± 0.10 | 1.23 ± 0.10 | 1.20 ± 0.11 | 1.16 ± 0.081 | n.s. |
|  | *LDLR/Tot Prot* | 0.056 ± 0.0071 | 0.059 ± 0.0071 | 0.052 ± 0.0072 | 0.048 ± 0.0067 | 0.050 ± 0.0070 | 0.043 ± 0.0061 | n.s. |
|  | *GR/Tot Prot* | 1.32 ± 0.20 | 1.37 ± 0.19 | 1.28 ± 0.20 | 1.29 ± 0.20 | 1.37 ± 0.22 | 1.32 ± 0.21 | n.s. |
|  | *LDLR/apoE* | 0.042 ± 0.0043 | 0.044 ± 0.0040 | 0.41 ± 0.0037 | 0.038 ± 0.0032 | 0.041 ± 0.0036 | 0.036 ± 0.0039 | n.s. |
|  | *GR/apoE* | 1.02 ± 0.13 | 1.05 ± 0.11 | 1.05 ± 0.13 | 1.07 ± 0.13 | 1.14 ± 0.13 | 1.16 ± 0.16 | n.s. |
| **mPFC** | *apoE/Tot Prot* | 0.65 ± 0.15 | 0.65 ± 0.13 | 0.67 ± 0.13 | 0.76 ± 0.18 | 0.57 ± 0.13 | 0.60 ± 0.13 | n.s. |
|  | *LDLR/Tot Prot* | 0.040 ± 0.0054 | 0.048 ± 0.0051 | 0.036 ± 0.0039 | 0.036 ± 0.0051 | 0.043 ± 0.0066 | 0.042 ± 0.0054 | n.s. |
|  | *GR/Tot Prot* | 0.21 ± 0.023 | 0.22 ± 0.019 | 0.22 ± 0.023 | 0.22 ± 0.025 | 0.23 ± 0.022 | 0.25 ± 0.029 | n.s. |
|  | *LDLR/apoE* | 0.12 ± 0.042 | 0.13 ± 0.045 | 0.10 ± 0.036 | 0.12 ± 0.052 | 0.15 ± 0.061 | 0.13 ± 0.049 | n.s. |
|  | *GR/apoE* | 0.60 ± 0.23 | 0.55 ± 0.16 | 0.53 ± 0.16 | 0.63 ± 0.24 | 0.66 ± 0.20 | 0.66 ± 0.17 | n.s. |
| **Liver** | *apoE/Tot Prot* | 0.40 ± 0.068 | 0.42 ± 0.081 | 0.31 ± 0.055 | 0.36 ± 0.066 | 0.36 ± 0.067 | 0.36 ± 0.061 | n.s. |
|  | *LDLR/Tot Prot* | 0.11 ± 0.020 | 0.12 ± 0.025 | 0.083 ± 0.016 | 0.10 ± 0.019 | 0.10 ± 0.017 | 0.10 ± 0.018 | n.s. |
|  | *LDLR/apoE* | 0.32 ± 0.046 | 0.35 ± 0.043 | 0.31 ± 0.036 | 0.30 ± 0.038 | 0.30 ± 0.029 | 0.34 ± 0.058 | n.s. |

**Supplementary Table 3** Nonsignificant Western blot results of apoE, LDLR, and GR for main effects of sex, genotype, and CVS. Values are shown as intensity normalized to total protein or apoE and hence have no units listed. *p* values refer to effects of genotype and CVS exposure; significance was *p <* 0.05.
