# Supplementary material for: Apolipoprotein E Isoform-specific changes related to stress and trauma exposure
Source: Transl Psychiatry. 2022 Mar 28;12:125. doi: 10.1038/s41398-022-01848-7 (PMC8960860; doi:10.1038/s41398-022-01848-7)
Supplement: Supplementary file 5 — Suppl. Table 4 [file 41398_2022_1848_MOESM5_ESM.docx]

**Supplementary Table 4** Additional sterol and oxysterol measures. Values are in ng/mg tissue, except cholesterol which is reported as ug/mg tissue. *p* values refer to effects of genotype and CVS exposure. Significance was set to *p* < 0.05.

| **Measure** | **E2** | | **E3** | | **E4** | | ***p*** |
| --- | --- | --- | --- | --- | --- | --- | --- |
|  | **Control** | **CVS** | **Control** | **CVS** | **Control** | **CVS** |  |
| **Hippocampus** | | | | | | | |
| Cholesterol | 6.79 ± 0.57 | 6.59 ± 0.85 | 7.40 ± 0.46 | 6.74 ± 0.34 | 6.90 ± 0.32 | 6.28 ± 0.96 | n.s. |
| Cholestanol | 22.34 ± 2.60 | 24.93 ± 1.59 | 26.40 ± 1.39 | 31.37 ± 1.06 | 24.38 ± 3.72 | 22.35 ± 2.91 | n.s. |
| Desmosterol | 126.50 ± 16.60 | 114.37 ± 25.07 | 122.27 ± 11.17 | 132.04 ± 11.17 | 108.36 ± 28.51 | 145.18 ± 38.66 | n.s. |
| Lathosterol | 14.46 ± 1.51 | 11.62 ± 2.77 | 13.10 ± 0.85 | 10.23 ± 0.75 | 9.55 ± 1.13 | 10.20 ± 1.20 | n.s. |
| **Cortex** | | | | | | | |
| 24S-hydroxycholesterol | 62.18 ± 25.46 | 71.81 ± 21.81 | 77.66 ± 24.50 | 101.07 ± 22.27 | 64.29 ± 15.98 | 88.35 ± 13.47 | n.s. |
| 25 hydroxycholesterol | 0.78 ± 0.14 | 0.60 ± 0.11 | 0.63 ± 0.11 | 0.68 ± 0.12 | 0.64 ± 0.047 | 0.61 ± 0.046 | n.s. |
| 27-hydroxycholesterol | 1.08 ± 0.089 | 0.99 ± 0.11 | 0.92 ± 0.12 | 0.97 ± 0.19 | 0.74 ± 0.12 | 0.90 ± 0.091 | n.s. |
