# Supplementary figures and images for: Apolipoprotein E Isoform-specific changes related to stress and trauma exposure
Source: Transl Psychiatry. 2022 Mar 28;12:125. doi: 10.1038/s41398-022-01848-7 (PMC8960860; doi:10.1038/s41398-022-01848-7)

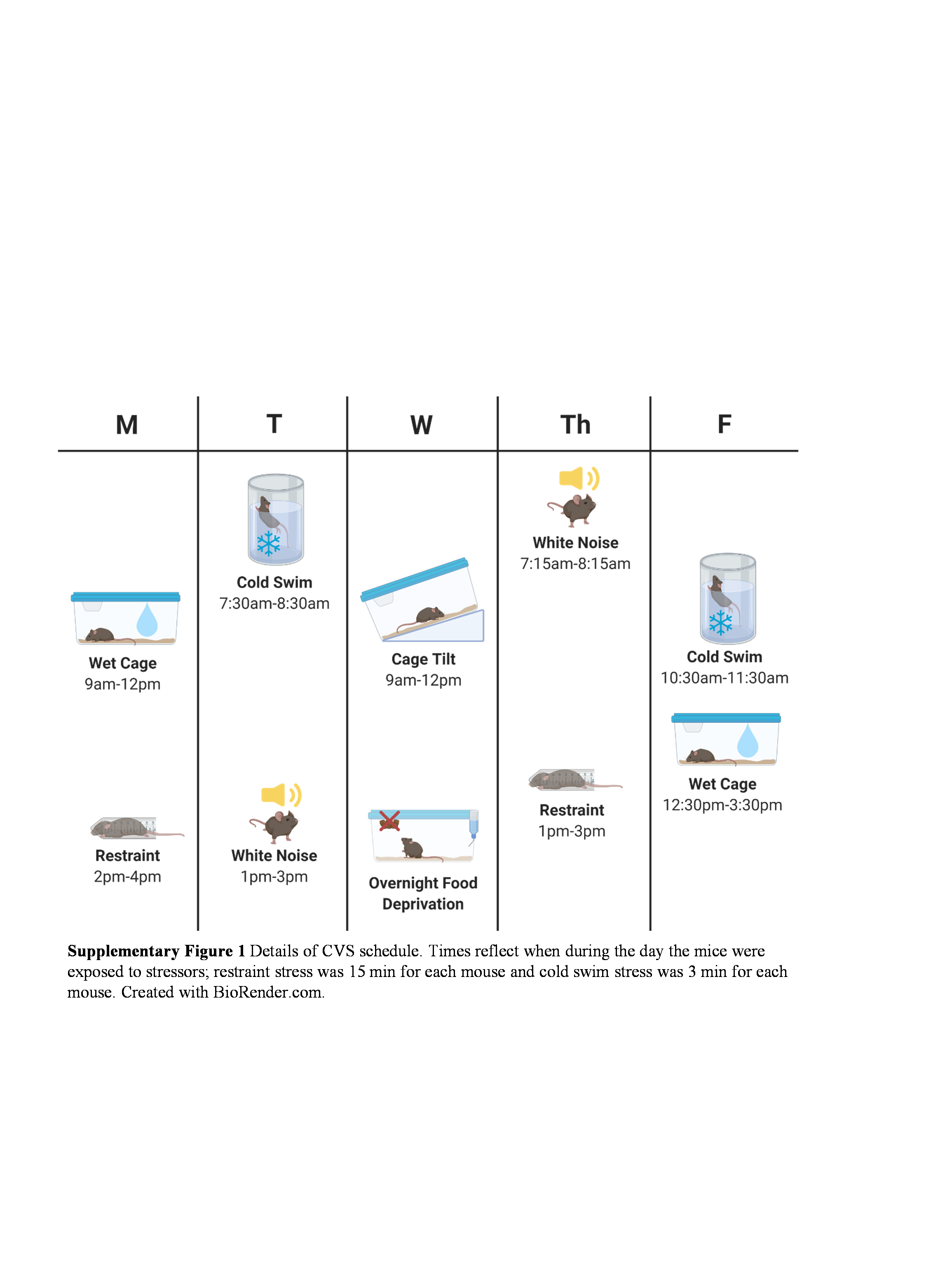

Supplement: Supplementary file 6 — Suppl. Figure 1 [file 41398_2022_1848_MOESM6_ESM.tif]

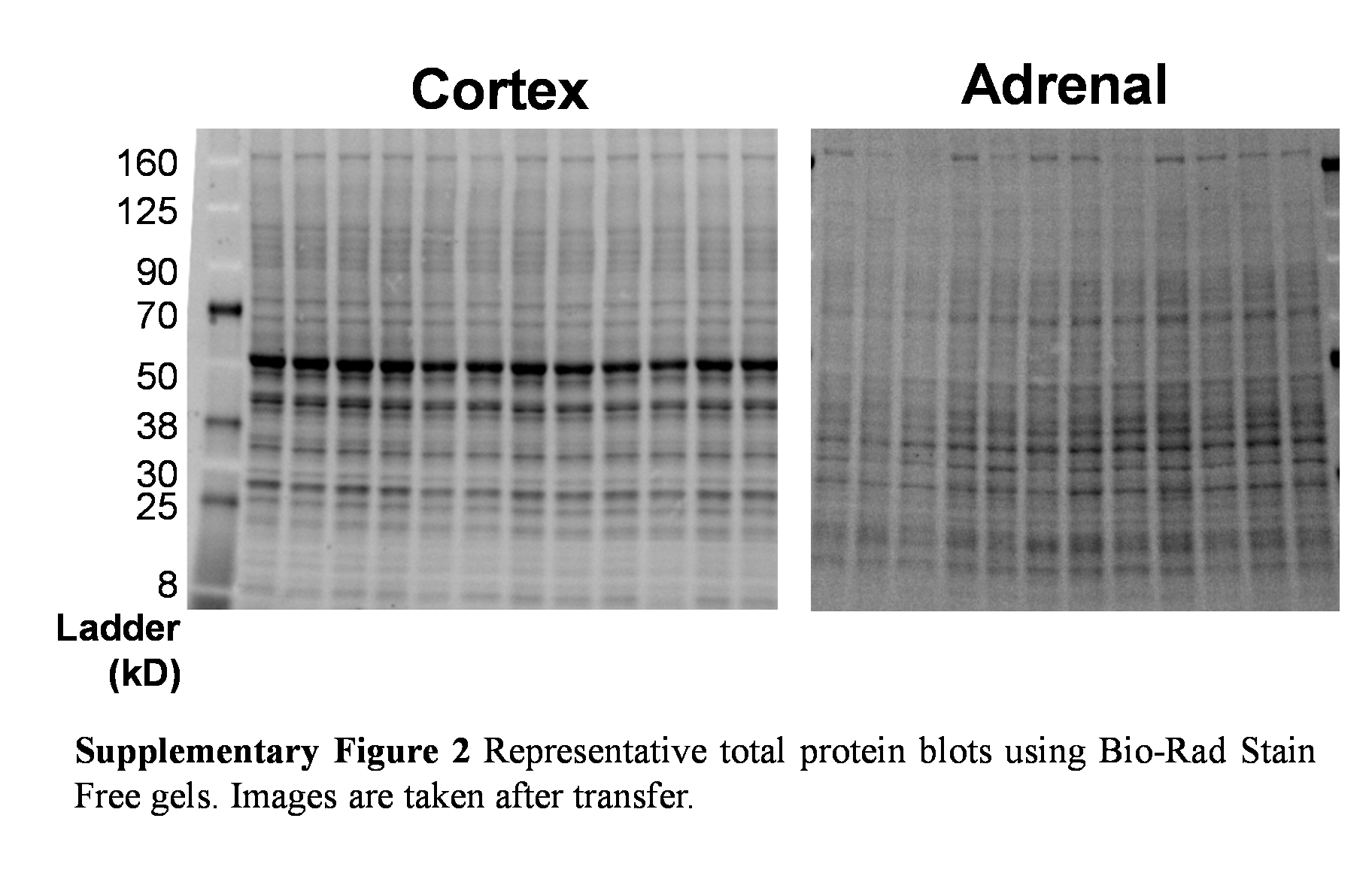

Supplement: Supplementary file 7 — Suppl. Figure 2 [file 41398_2022_1848_MOESM7_ESM.tif]

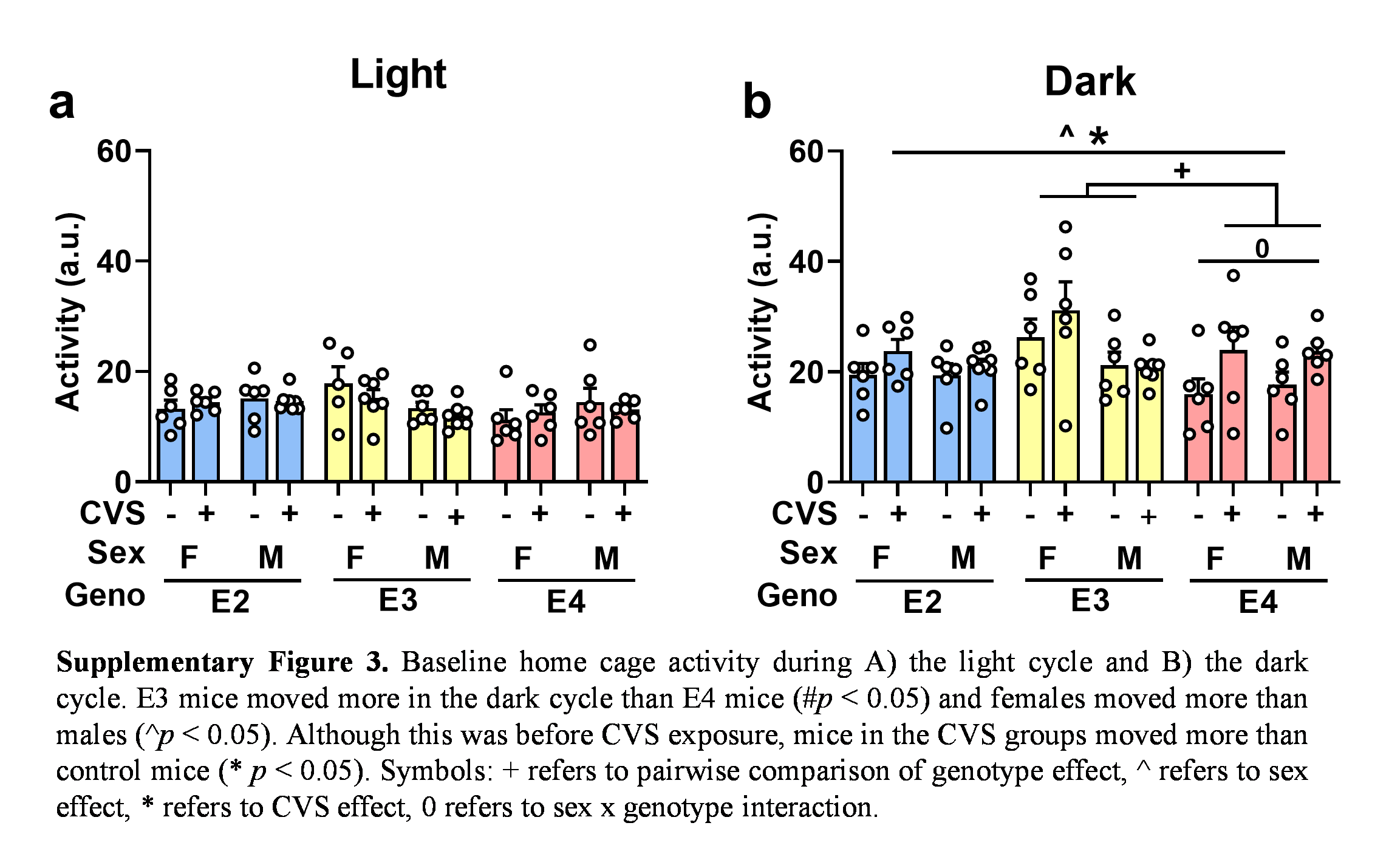

Supplement: Supplementary file 8 — Suppl. Figure 3 [file 41398_2022_1848_MOESM8_ESM.tif]

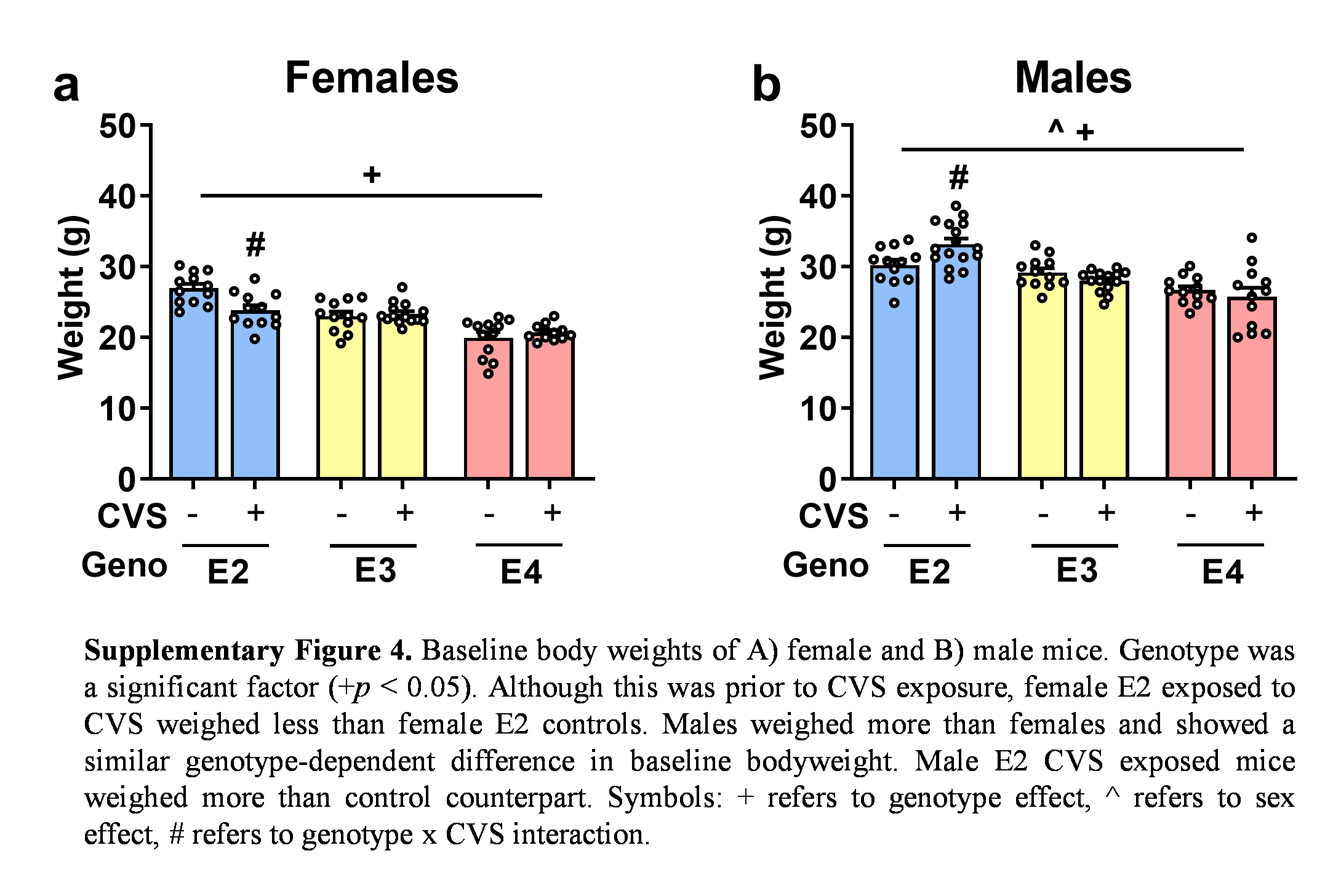

Supplement: Supplementary file 9 — Suppl. Figure 4 [file 41398_2022_1848_MOESM9_ESM.tif]
